# Multiomic study of skin, peripheral blood, and serum: is serum proteome a reflection of disease process at the end-organ level in systemic sclerosis?

**DOI:** 10.1186/s13075-021-02633-5

**Published:** 2021-10-15

**Authors:** Victor Farutin, Elma Kurtagic, Joël R. Pradines, Ishan Capila, Maureen D. Mayes, Minghua Wu, Anthony M. Manning, Shervin Assassi

**Affiliations:** 1 Momenta Pharmaceuticals Inc, Cambridge, MA USA; 2 Janssen Pharmaceutical Companies of Johnson & Johnson, 301 Binney St, Cambridge, MA 02142 USA; 3 Department of Medicine, Division of Rheumatology, The University of Texas Health Science Center at Houston, 6431 Fannin, MSB 5.270, Houston, TX 77030 USA

**Keywords:** Systemic sclerosis, Scleroderma, Gene expression, Proteomics

## Abstract

**Background:**

Serum proteins can be readily assessed during routine clinical care. However, it is unclear to what extent serum proteins reflect the molecular dysregulations of peripheral blood cells (PBCs) or affected end-organs in systemic sclerosis (SSc). We conducted a multiomic comparative analysis of SSc serum profile, PBC, and skin gene expression in concurrently collected samples.

**Methods:**

Global gene expression profiling was carried out in skin and PBC samples obtained from 49 SSc patients enrolled in the GENISOS observational cohort and 25 unaffected controls. Levels of 911 proteins were determined by Olink Proximity Extension Assay in concurrently collected serum samples.

**Results:**

Both SSc PBC and skin transcriptomes showed a prominent type I interferon signature. The examination of SSc serum profile revealed an upregulation of proteins involved in pro-fibrotic homing and extravasation, as well as extracellular matrix components/modulators. Notably, several soluble receptor proteins such as EGFR, ERBB2, ERBB3, VEGFR2, TGFBR3, and PDGF-Rα were downregulated. Thirty-nine proteins correlated with severity of SSc skin disease. The differential expression of serum protein in SSc vs. control comparison significantly correlated with the differential expression of corresponding transcripts in skin but not in PBCs. Moreover, the differentially expressed serum proteins were significantly more connected to the Well-Associated-Proteins in the skin than PBC gene expression dataset. The assessment of the concordance of between-sample similarities revealed that the molecular profile of serum proteins and skin gene expression data were significantly concordant in patients with SSc but not in healthy controls.

**Conclusions:**

SSc serum protein profile shows an upregulation of profibrotic cytokines and a downregulation of soluble EGF and other key receptors. Our multilevel comparative analysis indicates that the serum protein profile in SSc correlates more closely with molecular dysregulations of skin than PBCs and might serve as a reflection of disease severity at the end-organ level.

**Supplementary Information:**

The online version contains supplementary material available at 10.1186/s13075-021-02633-5.

## Background

Systemic sclerosis (SSc) is a complex, multisystem disease, characterized by an interplay of immune dysregulation, vasculopathy, and fibrosis [1]. Its clinical course is highly variable and reliable biomarkers that predict disease trajectory represent an area of unmet clinical need [2]. Samples from prominently affected fibrotic end-organs such as lung and skin are not typically obtained during SSc clinical care, while serum proteins are easily accessible because blood samples are routinely obtained as part of standard of care. However, it is unclear to what extent the SSc serum proteome is reflective of the molecular profile of peripheral blood cells (PBCs) versus affected end-organs, as studies that provide a direct, multilevel comparison between SSc serum profile, PBC, and skin transcriptome in concomitantly collected samples have not been reported.

Our recent study in the Scleroderma: Cyclophosphamide or Transplant Trial (SCOT) comparing the serum protein profile to PBC gene expression of enrolled patients in the concomitantly collected samples based on a panel of 230 measured proteins indicated that the differential expression of most serum proteins in SSc is likely to originate outside the PBCs [3]. However, skin biopsy samples were not obtained in this trial, and direct, multilevel comparison between serum proteome, PBC gene expression, and skin gene expression profiles could not be performed. In order to address this important knowledge gap, serum proteins using an extended panel of 911 analytes, as well as global PBC and skin gene expression profiles, were assessed in concomitantly collected samples of SSc patients and healthy controls. Subsequently, a multilevel comparison of generated molecular profiles was conducted employing three methodologies: (i) comparison of differentially expressed molecular profiles, (ii) assessment of a previously described approach that expands the differential expression analysis to include networks of connected proteins based on publicly available protein-protein-interaction data (Well-Associated-Protein [WAP] analysis [4]), and (iii) comparison of concordance of between sample similarities at the three investigated molecular levels [5]. These three analytic approaches showed concordant results indicating a stronger relationship between serum proteome and skin transcriptome than other comparisons (serum proteome vs. PBC transcriptome or PBC transcriptome vs. skin transcriptome) in SSc samples.

## Methods

### Patient selection

In this cross-sectional study, patients were recruited from the observational Genetic versus ENvironment In Scleroderma Outcome Study (GENISOS) cohort [6]. All patients fulfilled the ACR/EULAR Classification Criteria for SSc [7]. The extent of skin involvement was assessed by modified Rodnan Skin Score (mRSS) [8]. The mRSS assessments were performed by a rheumatologist with extensive experience with this skin thickness scoring approach (either MDM or SA). Clinically significant interstitial lung disease was defined as presence of high-resolution chest CT findings consistent with interstitial pulmonary involvement and a forced vital capacity of < 70%. Moreover, healthy controls of similar age- and racial/ethnic background were recruited. The healthy control participants did not have an autoimmune rheumatologic disease and were not first degree relative of patients with SSc. The study protocol was approved by the Institutional Review Board and all participants provided informed, voluntary consent.

### PBC gene expression profiling

PBC RNA collected in PAXgene tubes were obtained from the same participants included in our previously reported SSc skin gene expression study [9] at the time of skin biopsy. Total RNA was isolated according to the manufacturer’s protocol (PreAnalytiX blood miRNA kit). Similar to the skin gene expression study [9], global gene expression profiling was performed on Illumina HumanHT-12 BeadChip. PBC gene expression data has been deposited in the NCBI-GEO database (GSE179153).

### Matching skin gene expression profiling

As described previously, global gene expression profiling [9] was performed in matching punch skin biopsy samples obtained from the arm of study participants. These samples were immediately stored in RNAlater solution prior to RNA extraction. Global gene expression profiling was performed with Illumina HumanHT-12 BeadChip. The skin gene expression data have already been deposited in the GEO database (GSE58095).

### Serum proteomics by Olink PEA

A total of 981 proteins were assessed in concurrently collected serum samples by Olink Proximity Extension Assay (PEA) technology using 11 (cardiometabolic, cardiovascular—2 panels, cell regulation, development, immune response, inflammation, metabolism, neurology, oncology and organ damage) biomarker panels. Seventy proteins with more than 50% of observations below the lower limit of detection (LLOD) across the entire study cohort were excluded from the analyses (of note, there was no significant imbalance [at FDR < 5% level] in the proportion of samples below LLOD between SSc and Cont groups for these excluded proteins). For the remaining 911 unique serum proteins (see Additional file 2: Table S1 for the complete list of proteins), levels below the LLOD were replaced by the LLOD. Further details on pre-processing of Olink data are provided in the Additional file 1: Supplementary Materials.

### Data analysis

#### Pre-processing and normalization of molecular characterization data

Transcriptional profiling data for PBCs and skin has been limited to probes that have been mapped to Entrez Gene by current R/Bioconductor annotation and have average detection *p* value by Illumina below 0.01. Both gene and protein expression level data have been log base 2 transformed and quantile-quantile normalized prior to further analyses. Further details are provided in the Additional file 1: Supplementary Materials.

#### Differential expression analyses

Differential expression analysis of gene and protein levels were performed in R/Bioconductor [10, 11] using the “limma” framework [12, 13] to fit regression models adjusting for technical and biological covariates as further explained in the Additional file 1: Supplementary Materials. Multiple tests for statistical significance were adjusted for the number of comparisons based on the Benjamini-Hochberg procedure for estimating false discovery rate [14]. Statistically significant observations were defined as those with FDR < 5%.

#### Functional gene sets analyses

Gene signatures from the hallmark collection in the Molecular Signatures Database [15–17] were used to assess an overrepresentation of biological processes. Considering that both PBCs and skin included a complex set of different cell types, deconvolution analyses for the determination of cell type signatures were also pursued. For this purpose, the analytic approach recently developed by Uhlen et al. [18] was used for PBC gene expression dataset while the analytic approach by Swindell/Assassi et al. developed specifically for the skin transcriptome was utilized for the skin gene expression dataset [19, 20]. Statistical significance of gene set enrichment for differentially expressed genes was computed with limma-camera [21].

#### Direct comparison of differential expression in the serum protein dataset to the PBC and skin transcript datasets

The 911 proteins assessed by Olink were linked to their corresponding PBC and skin transcripts using Entrez gene IDs. Differentially expressed proteins/transcripts between SSc and controls were separately identified for each dataset as described in the Additional file 1: Supplementary Materials. Spearman’s rank-order correlation was calculated between differentially expressed proteins and PBC/skin transcripts in two separate analyses. These correlations were compared to a permutation-based ranking in which SSc/control status was assigned at random and resulting significance level was reported (for additional details, see Additional file 1: Supplementary Materials).

#### Assessing pathway connectedness between differentially expressed transcripts and proteins

The recently introduced WAP analysis [4] goes beyond differential gene expression analysis and incorporates prior knowledge about protein-protein interaction networks. This method was utilized to rank nodes (i.e. proteins) in the STRING network of protein-protein interactions [22] by their attachment to the more differentially expressed transcripts between SSc vs. control groups in the PBC and skin transcript datasets. The relationship between WAP scores in the skin and PBC transcript dataset for the differentially expressed serum proteins was investigated. (For additional details see Additional file 1: Supplementary Materials). Systematic difference between the ranks of the resulting WAP scores for the serum proteins (that are also differentially expressed in SSc vs. Cont comparison) as assessed for PBCs and skin transcript data would imply difference in their connectedness on pathway network to the SSc-Cont differences at transcriptome level in these two tissues. Ranks of WAP scores (within each tissue) were utilized rather than their actual values to alleviate the discrepancy in the magnitude of SSc-Cont differences in PBC and skin transcriptomic data. Comparison of WAP score ranks calculated for SSc-Cont differential expression in PBC and skin for the same set of proteins (such as those differentially expressed in serum) enables determination for which of these two tissues the selected proteins (irrespective of how it was selected) have higher connectedness on the network to the dysregulated transcripts in a given tissue. Edge-count probabilities [23] were utilized to evaluate significance of the number of edges observed between differentially expressed proteins and transcripts on the pathway network with respect to the null model of random graph with given expected degrees. Additional technical details related to these analyses are presented in the Section 6 of the Additional file 1: Supplementary Materials.

#### Assessment of between-samples similarities in PBCs, skin, and serum data

Correlation of similarities between the same set of samples characterized by two different sets of measurements (e.g., gene expression in PBCs and protein levels in serum) quantifies whether the samples that are more similar to each other by one set of measurements (e.g., PBC gene expression) are also more similar to each other in another measurement space (e.g., serum proteins). The advantage of this approach is that it can examine relationships between gene (or protein) modules even if they are composed of non-identical / non-overlapping genes (and/or proteins corresponding to them). A Mantel test [5]-based permutation procedure was employed to assess the concordance of between sample similarity across the datasets (serum proteome, PBC transcriptome, and skin transcriptome). Additional details on the implementation of Mantel test are provided in the Additional file 1: Supplementary Materials (Section 7).

## Results

### Clinical and demographic attributes

Table 1 summarizes key demographic and clinical attributes of patients with SSc (*n* = 49) and healthy controls of similar demographic background (*n* = 25) included in this multiomic study. The majority of patients had diffuse cutaneous involvement (65%) and a large portion of patients (40%) had clinically significant interstitial lung disease.

**Table 1:** Demographic and clinical characteristics of patients with SSc and healthy controls

	Cont	SSc	***p*** (SSc-Cont)	Diffuse	Limited	***p*** (Diff-Lim)
*N*	25	49		32	17	
Female, *N* (%)	21(84)	35 (71)	0.4^a^	21 (66)	14 (82)	0.37^a^
Age (years), mean (SD;IQR)	46(12;37-54)	52 (14; 46–62)	0.09^b^	49 (14; 43–58)	56 (13; 52–63)	0.08^b^
White, *N* (%)	14(56)	32 (65)	0.6^a^	19 (59)	13 (76)	0.38^a^
mRSS, mean (SD;IQR)	N/A	14 (11; 4.8–23)	N/A	18 (10; 9–26)	6.5 (6.1;3–6)	0.0001^b^
Disease duration (years), mean (SD;IQR)	N/A	7.8 (4.9;3.9–12)	N/A	6.3 (3.7;3.3–8)	10 (5.8; 5–15)	0.02^b^
Interstitial lung disease, *N* (%)	N/A	19 (40)	N/A	17 (57)	2 (12)	0.007^a^
Immunosuppressive agent, *N* (%)	N/A	14 (29)	N/A	10 (31)	4 (24)	0.81^a^
Anti-centromere Ab, *N* (%)	N/A	7 (14)	N/A	1 (3.1)	6 (35)	0.008^a^
Anti-topoisomerase Ab, *N* (%)	N/A	13 (27)	N/A	10 (31)	3 (18)	0.49^a^
RNA polymerase Ab, *N* (%)	N/A	12 (24)	N/A	11 (34)	1 (5.9)	0.06^a^
Ribonucleoprotein Ab, *N* (%)	N/A	3 (6.1)	N/A	2 (6.2)	1 (5.9)	1.0^a^

^a^By *χ*^2^ test

^b^By Wilcoxon-Mann-Whitney rank sum test

### Differential gene expression suggests increase in circulating innate immune cells in patients with systemic sclerosis

Comparison of SSc to control PBC gene expression profiles revealed 78 differentially expressed transcripts after correction for multiple comparisons (Additional file 2: Table S2). There were no differentially expressed genes when SSc patients with diffuse cutaneous involvement were compared to those with limited cutaneous involvement. Even though the SSc vs. control comparison revealed only a modest difference in average gene expression levels, it seemed to reflect blood cell types altered with disease. Genes that were reported by Uhlen et al. [18] as enhanced in immune cell types and/or lineages in blood are shown by column “UhlenCellTypeLineage” in Table S2 (Additional file 2). Genes with enhanced expression in granulocytes (including neutrophils and basophils) and monocytes were higher on average in SSc patients, while those with enhanced expression in B cells, T cells, and NK cells were on average lower in patients. Cumulatively, these results suggest potentially higher levels of immune cells representative of the innate compartment and, conversely, lower levels of those from the adaptive compartment in SSc PBC samples.

Next, a pathway analysis was carried out according to the hallmark collection of gene sets in MSigDB [15] which revealed interferon alpha and gamma response gene sets were by far the most significantly upregulated pathways in SSc vs. control comparison (Table 2).

**Table 2:** Overrepresented MSigDB hallmark signatures in SSc vs. control comparison in the PBC gene expression dataset

MSigDB ID	Size	Direction	PValue	%FDR	Description
M5911	75	Up	8E−13	4E−9	INTERFERON_ALPHA_RESPONSE
M5913	133	Up	9E−08	2E−4	INTERFERON_GAMMA_RESPONSE
M5932	96	Up	0.0003	0.6	INFLAMMATORY_RESPONSE
M5897	37	Up	0.0014	1.8	IL6_JAK_STAT3_SIGNALING
M5928	32	Down	0.0034	3.4	MYC_TARGETS_V2

In order to dissect which immune cell types might be modulated with disease, additional immune cell type deconvolution analysis according to Uhlen et al. [18] was performed using the entire PBC transcript dataset. Concordant with the above assessment of differentially expressed genes, this analysis revealed an upregulation of the neutrophil module and a downregulation of naïve CD4 and CD8 T cells in patients with SSc (Additional file 2: Table S3).

### Differential expression of gene sets in skin

A comparison of SSc to control skin global gene expression profile revealed 540 differentially expressed transcripts after correction for multiple comparisons. Pathway analysis of the MSigDB hallmark collection of gene sets (Additional file 2: Table S4) revealed epithelial-mesenchymal transition as the most significantly upregulated pathway in SSc skin samples followed by those for interferon alpha and gamma responses. Next, a previously described cell type signature analysis was employed [20]. As it was reported by Assassi et al. [19], fibroblasts, microvascular cells, and M2 macrophages were the most significantly enriched cell type signatures in SSc (Additional file 1: Figure S2b).

### Circulating proteins in SSc patients indicative of pro-fibrotic and pro-inflammatory processes

**Fig. 1:**
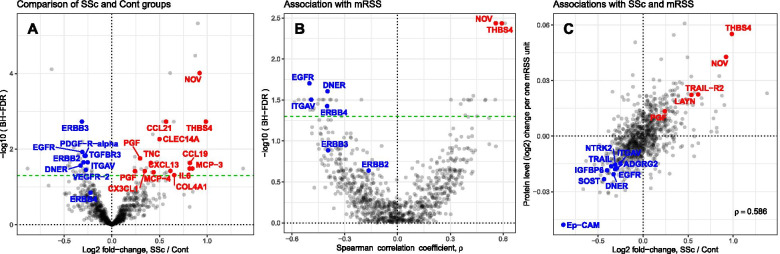
Serum proteins’ associations with disease and mRSS. **a** Volcano plot of SSc-Cont differences. **b** Volcano plot of correlation with mRSS in SSc patients. **c** Scatterplot of serum proteins associations with mRSS vs. SSc-Cont differences. Horizontal dashes (green) in **a** and **b** represent 5% FDR threshold. Text labels in **a** and **b** indicate proteins further discussed in the main text; in **c**—proteins associated at FDR < 5% both with mRSS and disease. Blue and red colors represent downregulation and upregulation respectively

**Fig. 2:**
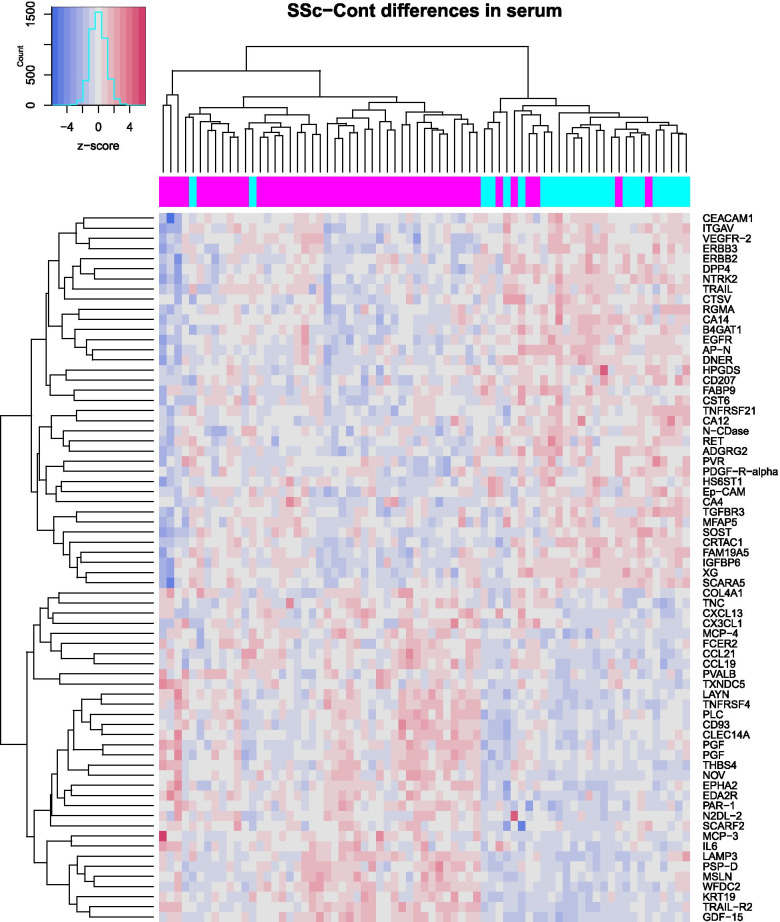
Heatmap of expression levels in the form of z-scores for the 70 serum proteins significantly different between SSc and Cont groups. Color bar above the heatmap indicates subjects from SSc (magenta) and Cont (cyan) groups

A concurrently collected serum sample was available in almost all samples (47 SSc and 24 healthy controls), and the proteomic profile of these serum samples was characterized by Olink technology resulting in assessment of 911 distinct proteins. Principal component analysis (PCA) plot (Additional file 1: Figure S1) indicated that SSc patients had a distinct serum protein profile in comparison to healthy controls (“multi-variate T” *p* < 0.001 [24, 25]). Assessment of differentially expressed proteins in SSc vs. Cont (Fig. 1a; Additional file 2: Table S5) after adjusting for age and gender yielded 70 unique proteins passing FDR < 5% cutoff. Table S6 in Additional file 2 provides the expanded (raw *p* < 0.05) list of serum proteins potentially reflecting SSc-Cont differences. As shown in Fig. 2, the list of upregulated (FDR < 5%) serum proteins included those involved in pro-fibrotic homing (IL-6, CLEC14A, TNC), extravasation (CX3CL1, CCL21, CCL19, CXCL13, MCP-3, MCP-4), and angiogenic pathways (PGF), as well as extracellular matrix (ECM) components/modulators (COL4A1, NOV, THBS4). Notably, several soluble growth factor receptors involved in fibrosis and vasculopathy were significantly downregulated (FDR < 5%) in SSc patients, including three epithelial growth factor receptors (EGFR, ERBB2, and ERBB3), VEGFR2 (the main receptor for VEGF, a key growth factor in angiogenesis), as well as TGFBR3 and PDGF-R-alpha (both key receptors in fibrotic response [26]). The remainder of serum proteins passing significance cutoff in this analysis, but lacking well established connections to SSc pathogenesis, might point at novel facets of biological processes in SSc and deserve further study, especially once reproduced by independent investigations.

### Association of serum proteins with mRSS

As shown in Fig. 1b and listed in Table 3, 39 proteins correlated significantly (FDR < 5%) with severity of skin involvement as assessed by mRSS. An expanded list of potential associations between serum protein levels and mRSS values (raw *p* < 0.05) is provided in Additional file 2: Table S7. The lower number of associations with mRSS passing 5% FDR cutoff, as compared to those associated with the differences between SSc patients and healthy controls, is likely due to the decreased statistical power for detecting associations with mRSS which was performed only for SSc patients and, therefore, for a smaller sample size. Text labels in Fig. 1c indicate 14 serum proteins passing 5% FDR both for their association with mRSS and SSc-Cont differences. Overall, 91 out of 95 proteins passing this cutoff for either of these two comparisons manifest concordant direction of their association with mRSS and with disease: proteins upregulated in SSc patients are also positively correlated with mRSS and, vice versa, negative correlation with mRSS for the proteins downregulated in SSc, suggesting their potential relevance for disease pathogenesis.

**Table 3:** Serum proteins significantly associated with modified Rodnan Skin Score (FDR < 0.05)

Protein name	Uniprot accession	***p***^a^	***ρ***^b^	***ρ***_**part**_^c^	SSc-Cont^d^
Cytokines					
CCL18	P55774	0.001	0.4	0.48	
GDF-8	O14793	0.002	− 0.32	− 0.45	
CCL3	P10147	0.001	0.3	0.31	
ECM proteins					
NOV	P48745	< 0.001	0.56	0.54	Up
SMOC2	Q9H3U7	< 0.001	0.55	0.51	
Enzymes					
CA6	P23280	< 0.001	− 0.42	− 0.48	
PCSK9	Q8NBP7	0.001	− 0.5	− 0.46	
DDC	P20711	0.002	− 0.38	− 0.37	
Growth factor receptors					
EGFR	P00533	< 0.001	− 0.5	− 0.54	Down
DNER	Q8NFT8	< 0.001	− 0.4	− 0.43	Down
ERBB4	Q15303	0.001	− 0.4	− 0.38	
NTRK2	Q16620	0.001	− 0.39	− 0.34	Down
Growth factors					
VEGFD	O43915	0.001	− 0.48	− 0.45	
PGF	P49763-3	0.002	0.45	0.39	Up
VEGFA	P15692	0.002	0.39	0.39	
Integrins					
ITGAV	P06756	< 0.001	− 0.49	− 0.47	Down
ITGB1	P05556	0.002	− 0.41	− 0.36	
Lectin					
Gal-4	P56470	0.001	− 0.53	− 0.52	
Others					
RCOR1	Q9UKL0	< 0.001	0.61	0.66	
THBS4	P35443	< 0.001	0.59	0.58	Up
PSIP1	O75475	< 0.001	0.56	0.55	
ALCAM	Q13740	0.001	− 0.59	− 0.54	
ADGRG2	Q8IZP9	0.001	− 0.56	− 0.54	Down
ENAH	Q8N8S7	0.001	0.5	0.5	
IGFBP6	P24592	0.002	− 0.5	− 0.5	Down
CD177	Q8N6Q3	0.001	0.42	0.47	
CD58	P19256	0.002	− 0.48	− 0.46	
SOST	Q9BQB4	< 0.001	− 0.38	− 0.46	Down
LYN	P07948	0.001	0.38	0.45	
DPP6	P42658	0.001	− 0.45	− 0.44	
Ep-CAM	P16422	0.001	− 0.45	− 0.44	Down
TF	P13726	0.001	− 0.43	− 0.43	
ZBTB17	Q13105	< 0.001	0.43	0.42	
LAYN	Q6UX15	0.001	0.41	0.41	Up
TRAIL	P50591	0.001	− 0.4	− 0.4	Down
IL-1RT2	P27930	0.001	− 0.35	− 0.39	
PECAM-1	P16284	0.001	− 0.21	− 0.31	
TNF receptor superfamily					
TRAIL-R2	O14763	0.001	0.5	0.49	Up
TNFRSF12A	Q9NP84	0.001	0.36	0.45	

^a^The significance of association from multiple linear regression model after adjustment for age and gender

^b^Spearman correlation between mRSS and protein expression

^c^Partial (adjusted for age and gender) Spearman correlation between mRSS and protein expression

^d^Direction of SSc-Cont differential expression (if significant)

Several serum proteins positively correlating with mRSS that were also upregulated in SSc vs. control comparison, include ECM proteins NOV and THBS4. Similarly, several proteins negatively correlating with mRSS were also significantly downregulated in SSc, such as EGF growth factor receptor (EGFR), EGF-related receptor DNER, and the integrin subunit alpha V (ITGAV). Overall, on the entire set of serum proteins, as shown in Fig. 1c and Additional file 1: Figure S3, the average differences between SSc and Cont groups were highly correlated (Spearman *ρ* = 0.59, permutation *p* < 0.0001; additional details can be found in Additional file 1: Supplementary Materials, Section 4) with their corresponding associations with mRSS (on SSc patients).

### Differential expression of serum proteins correlates significantly with the differential expression of corresponding skin transcripts in SSc vs. control

**Fig. 3:**
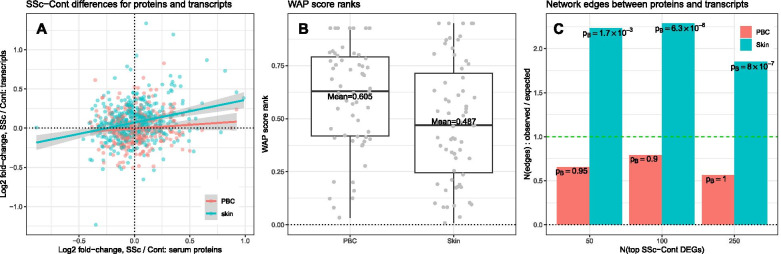
SSc-Cont differences for the serum proteins are significantly associated with SSc-Cont differences in skin. **a** SSc-Cont differences are positively correlated between serum proteins and corresponding skin transcripts. **b** WAP score ranks of differentially expressed serum proteins for SSc-Cont differences in skin and in PBC (lower values of rank represent more significant WAPs). **c** Ratios of observed to expected counts of pathway network connections between differentially expressed serum proteins and top 50, 100, and 250 most differentially expressed transcripts in SSc vs. Cont comparisons in skin (cyan) and in PBC (pink) and corresponding edge-count probabilities. Horizontal dashes (green) in **c** represent unremarkable case of the count of observed edges being equal to that expected for randomly rewired pathway network under null model of random graph with given expected degrees

In order to compare the SSc serum protein profile with the SSc skin and PBC transcript profiles, we first examined the correlation of serum protein differential expression in SSc vs. control comparison to the differential expression of corresponding transcripts in the examined gene expression datasets. Of the 911 proteins measured in serum, 314 had corresponding transcripts present in PBC, and 448 in skin gene expression data (Additional File 1, Section 5) that were included in this evaluation. The differential expression of serum protein significantly correlated with the differential expression of corresponding transcripts in the skin gene expression dataset (Spearman *ρ* = 0.21, permutation *p* = 0.012; Fig. 3a; Additional file 1: Figure S4) whereas a similar comparison between serum protein differential expression and PBC gene expression dataset yielded numerically lower rank correlation (Spearman *ρ* = 0.11) which did not reach statistical significance (permutation *p* = 0.25).

### Pathway network connectedness of serum proteins to PBC and skin transcripts

Differences in transcript levels between SSc and Cont were evaluated by WAP methodology in skin and PBC transcript datasets [4]. These analyses yielded two separate rankings of proteins based on the network of protein-protein interactions and the skin and PBC transcript data, as captured by their WAP scores. Lower ranks of WAP scores represent more pronounced connectedness on the pathway network to the more dysregulated transcripts between SSc and control groups in each transcript dataset. Comparison of the ranks of the WAP scores is intended to alleviate the impact of disparity in the magnitude of SSc vs. control differential expression between PBC and skin gene expression datasets. As shown in Fig. 3b, the 70 differentially expressed serum proteins in SSc vs. control comparison were ranked more prominently by WAP algorithm in the skin than in the PBC transcript datasets indicating higher network connectedness of these differentially expressed serum proteins to the SSc transcript dysregulations in the skin than those found in the PBCs. This increase in the network connectedness was significant by permutation analysis (*p* = 0.011) for the serum proteins with significant SSc-Cont differences, as well as for a wider range of serum proteins (Additional file 1: Figures S5a-b, S6).

Additionally, across a range of SSc-Cont differences at the transcript level (top 50, 100, and 250 transcripts with the lowest *p* values), differentially expressed serum proteins were several orders of magnitude more significantly connected to differentially expressed transcripts in skin as compared to PBCs (Fig. 3c, also see Additional file 1: Figure S5c). Cumulatively, these results reveal greater proximity on pathway network of serum proteins and skin transcripts perturbed in SSc (as compared to those in PBCs) and suggest that the SSc serum protein profile is reflective of the dysregulations at the skin level.

### Correlation of similarities between serum proteins and skin and PBC transcriptional profiles suggests disruption of cell type homeostasis with disease

Transcriptional profiles of PBCs and skin biopsies obtained concomitantly with serum proteomic data for the same SSc patients and healthy controls enable assessment of correlation between these molecular measurements at the level of individual genes/proteins, as well as the assessment of the concordance of between-sample similarities among these three data levels.

A comparison of corresponding transcripts between skin and PBC transcriptomes revealed that the expression levels of 105 transcripts significantly correlated (FDR < 5%) in these two tissues types in SSc and/or control samples. As shown in Additional file 1: Figures S7-S11 and listed in Additional file 2: Table S8, the correlations were predominantly positive and concordant in patients and controls. Some of the biological themes, prominently represented by the genes positively correlating between skin and PBC transcriptome in SSc patients and controls, included genes encoding ribosomal proteins (e.g., RPL14, RPS12, RPS26 and RPS23), interferon-inducible proteins (e.g., IFI27, MX1, OAS2 and HERC5), and HLA class I (HLA-A, HLA-C and HLA-H) and II (HLA-DPB1, HLA-DQB1, HLA-DRB1 and HLA-DRB4). There were also few transcripts in which the direction of skin-PBC correlation was discordant in SSc and healthy control samples (TCHP, TRPT1, NFKBIA, and MT1X).

**Fig. 4:**
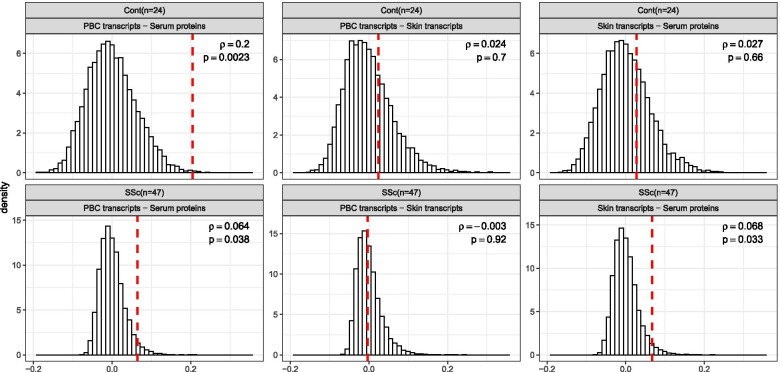
Concordance of between samples similarities (as Spearman correlations) for each pairwise combination of the three datasets: serum proteins, PBC transcripts, and skin transcripts. Top row displays results for healthy controls, bottom row—for SSc patients. Mantel test results (vertical red dashes represent the observed concordance of between sample similarities for the actual mapping of samples to subjects) are compared to their corresponding null distributions (obtained by randomly permuting assignment of samples to subjects in each dataset)

Lastly, we focused on the concordance of between-samples similarities at the entire dataset level. Of note, we anticipated a priori that the correlations in this comparison will be weaker than the aforementioned methods because this comparison goes beyond differentially expressed molecules and examines the entire dataset that includes many genes/proteins which are unaltered in the disease state, including housekeeping genes/proteins. However, such agnostic evaluation across all analytes characterized for each pairwise comparison eliminates the potential of introducing the bias associated with variable selection based on intensity, variability or differential expression. Figure 4 represents results of pairwise comparisons of between-samples similarities in the entire PBC transcript, skin transcript, and serum protein datasets for SSc patients and healthy controls (vertical red dashes) in comparison to null distributions of those metrics obtained by random matching of molecular profiles for study subjects (represented as histograms). The most significant concordance of between-samples similarities is observed between PBC gene expression data and serum proteins in healthy controls (*ρ* = 0.2, *p* = 0.002). This suggests strong influence of PBC transcriptional profile onto the levels of circulating proteins in serum for healthy controls. The correlation of similarities between PBC and skin transcriptional profiles for the healthy controls was not significant (*ρ* = 0.02, *p* = 0.7) and comparable to the correlation observed between serum proteome and skin transcriptional profile observed in the same study group (*ρ* = 0.03, *p* = 0.66 for healthy controls). These two observations taken together suggest that, for healthy controls, the genome-wide similarity of skin transcriptional levels has very little, if any, relevance to the genome-wide similarity of PBC transcriptional levels and to serum protein level similarities. Conversely, the correlation of similarities between molecular profiles of PBC transcriptome and serum proteins in SSc patients is lower in magnitude (*ρ* = 0.06, *p* = 0.04) compared to correlation between the same compartments in healthy controls. This correlation in SSc patients is also similar to that observed between skin transcriptome profile and serum protein in the same study group (*ρ* = 0.07, *p* = 0.03). Both correlations are statistically significant with respect to permutation, indicative of the comparable impacts of both skin and PBC transcriptional composition on the levels of circulating proteins in serum of SSc patients. The similarities among gene expression profiles in skin and in PBCs in SSc patients showed the weakest correlation (*ρ* = − 0.003, *p* = 0.9) among all six performed comparisons.

## Discussion

In the present study, the comparison of skin and PBC transcriptomic data to serum protein profile in concurrently collected samples examining differential expression, WAP analysis, and overall between-sample concordance showed consistently that the serum proteome reflects molecular dysregulation in the skin tissue, in SSc patients. These results are consistent with our previous findings in the baseline line samples of the SCOT study, in which only a small portion of differentially expressed serum proteins (15.5%) was also differentially expressed in the concurrently collected PBC transcriptome, supporting the notion that differential expression for most serum proteins in SSc is likely to originate outside the PBCs. This finding might be counterintuitive as PBCs and serum proteins are proximally located in the intravascular compartment. The correlative analysis of between sample similarities in the present study indicates that the correlation between the PBC gene expression profile and serum proteome in SSc patients, contrary to healthy controls, is weakened by the spillover effect of molecular dysregulation in the skin tissue. The observed prominent correlations between serum proteins and the extent of SSc skin involvement as assessed by mRSS further supports a link between molecular dysregulation at the serum protein and skin levels in SSc. Consistent with our results, two previous proteomic studies have also shown a large number of serum proteins correlating with mRSS in patients with diffuse cutaneous involvement [3, 27]. Cumulatively, these results indicate that serum proteins are attractive surrogate markers for tracking disease severity at the diseased organ level.

In the present study, the Olink platform enabled an interrogation of a large number of serum proteins across broad variety of cardiovascular, metabolic, inflammatory, immune, developmental, neurological, and carcinogenic pathways. Focusing on the biological pathways, one of the notable findings was the downregulation of several soluble growth factor receptors involved in fibrosis including four EGF receptors (EGFR, ERBB2, ERBB3, and ERBB4). EGFR also showed a strong negative correlation with mRSS. These findings are consistent with findings in the SCOT cohort in which soluble EGFR was significantly downregulated in SSc and showed the strongest negative correlation with mRSS [3]. Decrease in circulating soluble EGFR has been previously described in malignancies [28]. Specifically, soluble form of EGFR can sequester EGF ligand, preventing it from binding and activating membrane bound EGFR [29]. Overall, a downregulation of soluble EGF receptors in SSc patients in our study might imply general upregulation of EGF receptor pathways. EGF signaling has been implicated in the pathogenesis of pulmonary and renal fibrosis [30–37], but only little evidence exists for skin fibrosis. One study reported that SSc-derived PDGFR autoantibodies can induce profibrotic effects in vitro, through transactivation of the EGFR [38]. Moreover, aberrant activation of EGF-mediated signaling pathways in dermal fibroblasts can lead to the upregulation of TGFBRII, TGFβ receptor, which is a prominent profibrotic mediator [39]. A more recent multi-cohort analysis of SSc skin transcriptome data across 7 datasets composed of 515 samples identified 6 positively correlated signaling proteins for the SSc transcript signature, four of which were EGFR ligands [40]. Our study provides additional evidence for potential involvement of EGF receptor family members in SSc pathogenesis. Furthermore, strong negative correlation of soluble EGF receptor family members with mRSS warrants exploration of their expression in longitudinal patient samples and their potential as biomarkers. While we observed decreased level of several soluble profibrotic growth factor receptors such as TGFBR3 and PDGFR-alpha in SSc serum, a previous study has indicated an increased level of N-terminal connective tissue growth factor (CTGF) in SSc plasma [41], indicating that profibrotic growth factor levels might be increased in SSc serum while the soluble receptor levels of profibrotic growth factors are low. This finding might be due to decreased shedding of these receptors in the fibrotic tissue.

The number of differentially expressed transcripts in the PBC in the present study was lower than previously observed in patients with early diffuse disease with severe internal organ involvement in the SCOT study [42]. However, we and others have observed similar number of differentially expressed genes in SSc PBCs in more representative patient samples [43, 44]. In order to account for the fact that the SSc gene expression profile in skin is more distinct than in PBCs in comparison to healthy controls, we complemented the comparison of differentially expressed transcripts/proteins across the three tissue types by assessment of networks of connected proteins (WAPs) and global concordance analysis of between sample similarities.

In relation to SSc pathophysiology, consistently with previously published data [43–49], interferon response pathways were among the top upregulated pathways in both SSc PBC and skin transcriptome in the present study. Notably, several prominent IFN inducible genes (IFI27, MX1, OAS2, and HERC5) were among a limited number of transcripts whose expression in the PBC transcriptome directly correlated with their expression in the skin tissue, indicating that there is a biological link between the IFN signature in the PBCs and disease affected tissue in SSc. This finding is consistent with the previously reported strong correlation of the IFN gene expression signature in PBCs and disease affected tissue in systemic lupus erythematosus (skin), dermatomyositis (muscle), and SSc (skin) [50].

The present study has several strengths. To our knowledge, it represents the first, multi-level examination of serum proteome, PBC, and skin gene expression data in concurrently collected samples in patients with SSc. Furthermore, the utilized proteomic platform enabled reliable assessment of a large panel of serum proteins involved in various disease processes. Moreover, the utilized analytic approach goes beyond assessment of differentially expressed proteins and included examination of networks of connected proteins and concordance of between sample similarities. As a result, we have provided three lines of evidence supporting the plausibility of serum proteome reflecting disease process at the end-organ level in SSc: (1) globally, the correlation of the differences between SSc and Cont is more pronounced for serum proteins and corresponding skin, rather than PBC transcripts; (2) serum proteins differentially expressed in SSc are more significantly connected on the pathway network to the skin, than to PBC transcripts dysregulated in disease; and (3) overall concordance of between-subject similarities across the entire serum protein and skin transcript datasets is more pronounced in SSc patients than in healthy controls.

However, our study also has some limitations. While it is limited to cross-sectional samples and does not enable evaluation of the longitudinal aspect of SSs pathogenesis, future studies can longitudinally investigate the relationship between PBC, skin, and serum molecular profiles in SSc patients. Additionally, although we used a large-scale, robust platform, comparisons involving serum proteins were limited to the proteins included in Olink PEA panels and could be potentially impacted by expanding these analyses to a wider range of serum proteins. Moreover, the present study was not confined to patients with early diffuse disease, molecular characterization of patients with early severe disease in similar manner represents an exciting possibility that can be pursued in future studies. However, our results are in agreement with the PBC gene expression and serum protein comparative analysis in the SCOT trial which included only patients with early diffuse cutaneous involvement [3].

## Conclusions

In conclusion, our study expands the findings of previous reports of the upregulated profibrotic cytokines and downregulated soluble EGF and other key receptors in serum proteome of SSc patients. Furthermore, SSc PBC and skin transcriptome both showed a prominent type I IFN signature. Most notably, the present study represents the first, multi-level examination of serum proteome, PBC, and skin gene expression data in concurrently collected samples in patients with SSc. This enabled a direct comparison of these three sample types and revealed that the primary contributor to SSc serum protein profile is diseased tissue rather than PBCs. This finding underscores the potential utility of serum proteins as attractive surrogate markers for tracking disease severity at the diseased organ level in SSc.

## Supplementary Information

**Additional file 1.** Supplementary Materials; Figures S1-S11.

**Additional file 2.** Tables S1-S8.

## Data Availability

The datasets generated and/or analyzed during the current study are available in the NCBI-GEO repository under the following accession numbers: GSE58095, GSE179153

## Abbreviations

CCL19: C-C motif chemokine ligand 19
CCL21: C-C motif chemokine ligand 21
CCN3: Cellular communication network factor 3
CLEC14A: C-type lectin domain containing 14A
COL4A1: Collagen type IV alpha 1 chain
CX3CL1: C-X3-C motif chemokine ligand 1
CXCL13: C-X-C motif chemokine ligand 13
DNER: Delta/notch like EGF repeat containing
ECM: Extracellular matrix
EGFR: Epidermal growth factor receptor
ERBB2: erb-b2 receptor tyrosine kinase 2
ERBB3: erb-b2 receptor tyrosine kinase 3
FDR: Benjamini-Hochberg false discovery rate
GENISOS: Genetics versus Environment in Scleroderma Outcomes Study
HERC5: HECT and RLD domain containing E3 ubiquitin protein ligase 5
IFI27: Interferon alpha inducible protein 27
IL-6: Interleukin 6
ITGAV: Integrin subunit alpha V
LLOD: Lower limit of detection
MCP-3: Monocyte chemotactic protein 3
MCP-4: Monocyte chemotactic protein 4
mRSS: Modified Rodnan Skin Score
MSigDB: Molecular Signatures Database
MX1: MX dynamin like GTPase 1
NOV: Nephroblastoma overexpressed (CCN3)
OAS2: 2′-5′-oligoadenylate synthetase 2
PBC: Peripheral blood cells
PDGF-Rα: Platelet-derived growth factor receptor alpha
PGF: Placental growth factor
SCOT: Scleroderma: Cyclophosphamide or Transplant trial
SSc: Systemic sclerosis
TGFBR3: Transforming growth factor beta receptor 3
THBS4: Thrombospondin 4
TNC: Tenascin C
VEGFR2: Vascular endothelial growth factor receptor 2
WAP: Well-Associated-Protein
