# Supplementary material for: Multiomic study of skin, peripheral blood, and serum: is serum proteome a reflection of disease process at the end-organ level in systemic sclerosis?
Source: Arthritis Res Ther. 2021 Oct 15;23:259. doi: 10.1186/s13075-021-02633-5 (PMC8518248; doi:10.1186/s13075-021-02633-5)
Supplement: Supplementary file 1 — Additional file 1. Supplementary Materials; Figures S1-S11. [file 13075_2021_2633_MOESM1_ESM.pdf]

Multiomic study of skin, peripheral blood and serum: Is serum proteome a reflection of disease process at the end-organ level in systemic sclerosis?

Supplementary Material

Victor Farutin, Elma Kurtagic, Joel R. Pradines, Ishan Capila, Maureen D. Mayes, Minghua Wu, Anthony M. Manning, Shervin Assassi

30 August, 2021

Contents

1 Data pre-processing and normalization **2**

1.1 Peripheral blood cells (PBC) and skin transcriptomics data 2

1.2 Serum proteomics data 2

2 Differential expression analyses **2**

2.1 PBC transcripts 2

2.2 Skin transcripts 3

2.3 Serum proteins 3

3 Functional gene sets analyses **3**

4 Correlation between serum proteins SSc-Cont differences and associations with mRSS **5**

5 Comparison of SSc-Cont differential expression in the serum, PBC and skin **6**

6 Pathway connectedness between differentially expressed transcripts and proteins **7**

7 Comparison of similarities among molecular profiles of skin, PBC and serum **8**

7.1 Correlations between transcripts in PBC and skin and serum proteins 8

7.2 Mantel test-based evaluation of the concordances of between-sample similarities 10

Session Info **16**

Supplementary References **16**

1 Data pre-processing and normalization

1.1 Peripheral blood cells (PBC) and skin transcriptomics data

For differential expression and gene sets analyses, probes on Illumina chip were quantile-quantile normalized and restricted to the overlap between probes mapped to Entrez Gene (by "re-annotated" mapping in Bioconductor package `illuminaHumanv4.db`) and those with average detection p-value equal to or below 0.01. This resulted in 8486 and 11832 probes for PBC and skin gene expression datasets respectively. The multiple regression linear model used for differential gene expression with `limma::lmFit` (and gene set analysis by `limma::camera`) included Illumina chip identifier (Sentrix_ID, as categorical attribute) and sample collection date (as continuous covariate) to account for their impact on PBC and skin gene expression data. Regression models for differential expression analyses of serum proteins (for the differences between SSc and Cont groups and for the association with mRSS within SSc patients) included subject age and gender as continuous and categorical covariates, respectively. For gene set analyses, when multiple Illumina probes were mapped to the same gene identifier, the average of their normalized expression were used. Such gene-level transcriptional datasets had 6411 and 8978 genes characterized for PBC and skin samples respectively.

1.2 Serum proteomics data

Selected protein levels in serum samples from 71 subjects in this study were measured using Olink Proximity Extension Assay (PEA) technology. Protein expression levels in serum samples as measured by Olink PEA have been filtered to remove proteins that predominantly (>50% of the samples) measured at or below lower limit of detection (LLOD). For the remainder of the proteins, levels measured at or below LLOD were replaced by the corresponding LLOD values. Small fraction (~0.1%) of otherwise missing values (e.g. due to sample failure for a given panel) were replaced with their average values for corresponding protein assays. Profiles of the proteins characterized by more than one panel (e.g., IL-6 is included in four panels) were highly correlated between the panels (Pearson r IQR=0.94-0.99). For these proteins profiles for each of the panels were replaced with average values across all panels, resulting in 911 distinct proteins used for further analyses. Normalized Protein eXpression (NPX) values for these proteins were quantile-quantile normalized prior to the unsupervised learning and differential expression analyses presented below.

2 Differential expression analyses

2.1 PBC transcripts

Supplementary Table S2 in the supplementary data file `Additional_File_2.xlsx` displays 78 Illumina probes (together with corresponding gene annotation) that pass BH-FDR threshold of 0.05 for their association with the differences between gene expression levels in PBC samples from study subjects with systemic sclerosis (SSc) and healthy controls (Cont) as estimated by `limma` model accounting for the effects of Illumina chip identity and sample collection date. Additionally, 437 and 1216 Illumina probes pass BH-FDR cutoffs of 10% and 20% respectively. Constraining comparison between healthy controls and SSc patients only to those with diffuse disease resulted in a larger number of statistically significant differences (e.g., 162 with BH-FDR < 0.05) and high genomewide correlation (Spearman $\rho = 0.973$) to the differences observed between healthy controls and the entire group of SSc patients (both with diffuse and limited disease). Multiple regression analysis performed on SSc samples evaluating effect of disease duration on gene expression levels adjusting for the effects of age as well as sample collection date and Illumina chip identity did not detect statistically significant associations with disease duration in transcriptional profiling data in PBC (BH-FDR > 0.22). No statistically significant differences were detected between SSc patients on immunosuppressive agents and those not undergoing this treatment (BH-FDR > 0.37). Main text Table 2 displays "hallmark" MSigDB gene sets that pass BH-FDR<0.05 cutoff in `limma::camera` analysis of SSc-Cont differences additionally accounting for the effects of technical covariates in the study.

2.2 Skin transcripts

Differential analysis of SSc-Cont comparison using skin transcript data identified 540 Illumina probes that pass BH-FDR threshold of 0.05. Application of more relaxed cutoffs on BH-FDR of 10% and 20% yields 1273 and 3002 Illumina probes respectively associated with the differences between SSc and Cont groups in skin. Similarly to the PBC transcripts, differences in skin gene expression data between healthy controls and SSc patients with diffuse disease were highly correlated (Spearman $\rho = 0.97$ genomewide) to the differences between healthy controls and all SSc patients (with diffuse and limited disease) and manifested higher statistical significance (1117 Illumina probes passed BH-FDR < 0.05 cutoff). Supplementary Table S4 in the supplementary data file `Additional_File_2.xlsx` displays "hallmark" MSigDB gene sets that pass BH-FDR<0.05 cutoff in `limma::camera` analysis of SSc-Cont differences in skin upon adjusting for the effects of sample collection date and Illumina chip identity.

2.3 Serum proteins

Supplementary Figure S1 displays two-dimensional projection of this data using principal components analysis (PCA) performed on protein-wise centered and scaled NPX values. Color and shape of the symbols in the plot indicate healthy controls (red dots) and patients with systemic sclerosis (blue triangles) and this suggests difference between the two groups. Significance of the differences between two groups of observations in multiple dimensions (e.g. difference between healthy controls and patients with SSc across the entire set of serum proteomics readouts) was assessed using a "multi-variate T" (MVT) approach (1,2). Serum proteomic profiles by Olink PEA are systematically different between SSc and Cont subjects in this study (MVT p-value 10^{-4}).

Associations between serum protein levels as measured by Olink and SSc-Cont differences and mRSS (within SSc group) were evaluated with multiple regression model adjusting for the effects of gender and age of study subjects. Gender and age were found (at BH-FDR<5% level) to impact 13 and 59 serum proteins, respectively. Supplementary Table S5 in the supplementary data file `Additional_File_2.xlsx` and main text Table 3 display associations of serum protein levels at BH-FDR<5% with SSc-Cont differences and mRSS (within SSc patients), respectively. Supplementary Tables S6 and S7 provide expanded (unadjusted p<0.05) tables of these results. As reported above for PBC and skin transcripts, comparison of SSc patients with diffuse disease only to healthy controls yielded larger number of differentially expressed proteins (120 with BH-FDR < 0.05) that were highly correlated to the differences between all SSc patients (both with limited and diffuse disease) and healthy controls (Spearman $\rho = 0.958$ across all proteins characterized in serum). Regression modeling that involved mRSS attribute that is defined only for patients with systemic sclerosis was performed for the subset of 46 patients for which this attribute is represented. All continuous attributes have been centered for the purposes of regression analysis. As suggested by PCA and MVT results described above, a substantial fraction of serum proteins measured by Olink passes statistical significance threshold of BH-FDR<0.05 for their association with SSc-Cont differences. Main text Figure 2 displays their expression levels in the form of a heatmap.

3 Functional gene sets analyses

Analysis of functional gene sets for their enrichment for the differences between SSc patients and healthy controls was performed using `limma::camera` (3). The gene sets that were recently reported by Uhlen et al. (4) as representative of immune cell types in blood were restricted to the genes specific to particular cell types (i.e. by excluding those genes that were reported by Uhlen et al. as enhanced in more than one cell type) and used for evaluating SSc-Cont differences in PBC data. Gene sets representative of cell types in skin (5) were scored for their association with SSc-Cont differences in gene expression data from skin biopsies matched on study subjects (6). Gene sets specific to immune cell types in blood that pass significance cutoff (BH-FDR<0.05) for their association with SSc-Cont differences in PBC are shown in the Supplementary Table S3 in the supplementary data file `Additional_File_2.xlsx`. Supplementary Figure S2 depicts in the

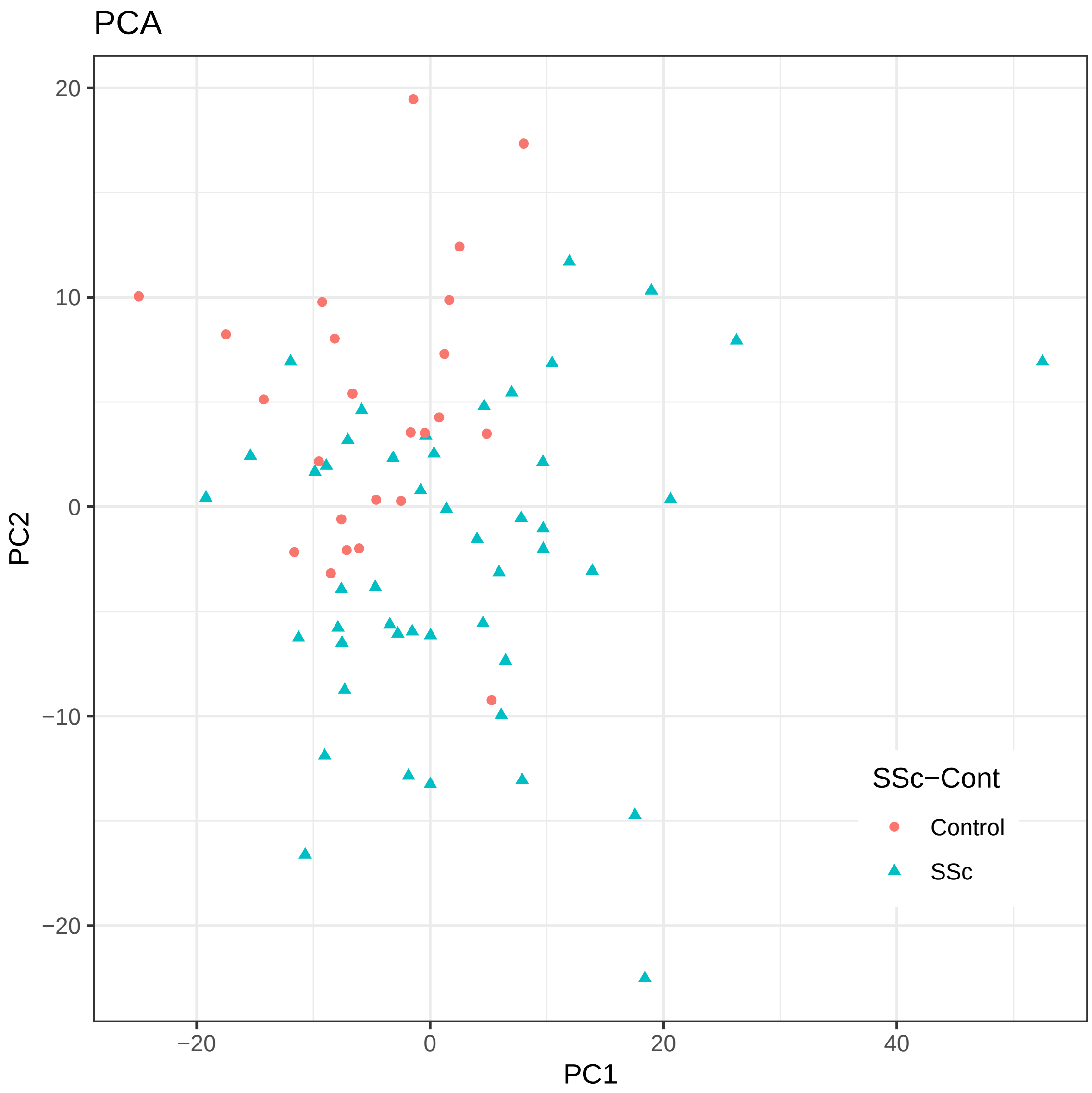

Figure S1: Principal components analysis (PCA) representation of the healthy controls (Cont) and study subjects with systemic sclerosis (SSc) in the space of Olink PEA NPX measurements. Colour and shape of the symbols indicate SSc-Cont status of study subjects.

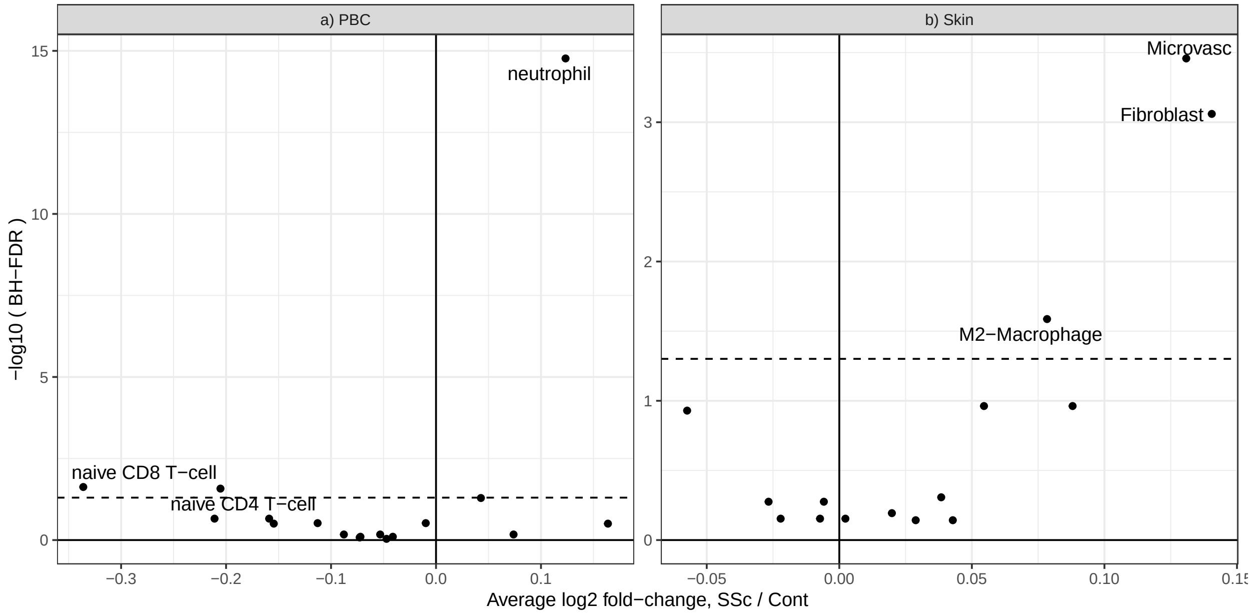

Figure S2: Significance (as negative log base 10 of BH-FDR) vs. average SSc-Cont differences (on log base 2 scale) for cell type specific gene sets in skin and PBC gene expression data.

form of volcano plots the results of scoring cell type gene sets in PBC (panel a) and skin (panel b) for their enrichment with SSc-Cont differences in concordant direction comparatively to the rest of the genes included in the analysis.

Main text Table 2 and Supplementary Tables S3 and S4 display significantly associated MSigDB hallmark gene sets as well as those representative of cell types in PBC and skin. Besides gene set descriptions and identifiers these tables also include count of genes in the gene set with at least one Illumina probe mapped to them (column "Size"), average SSc-Cont difference for the gene set (column "Direction": "Up" corresponds to upregulation in SSc as compared to Cont) as well as statistical significance and BH-FDR (columns "PValue" and "FDR" respectively).

For the transcriptional profiles of PBC samples (Supplementary Figure S2, panel a), genes that are specifically enhanced in neutrophils are by far the most significantly enriched for positive SSc-Cont differences, suggesting their average increase in PBC samples from SSc as compared to Cont group. Gene sets representative of naive CD4 and CD8 T-cells also pass BH-FDR<0.05 cutoff and are on average decreased in SSc as compared to Cont PBC samples. Consistently with the results reported earlier in (6), evaluation of gene sets representative of cell types in skin for their enrichment with SSc-Cont differences performed herein (Supplementary Figure S2, panel b) identified gene sets for fibroblasts, M2 macrophages and microvascular cells as the most significantly enriched for genes with average increase in their expression levels in SSc (as compared to Cont) samples.

4 Correlation between serum proteins SSc-Cont differences and associations with mRSS

Overall, on the entire set of serum proteins, the average differences between SSc and Cont groups are positively correlated (Spearman $\rho = 0.586$) with their corresponding associations with mRSS on SSc patients indicating that proteins elevated in the SSc patients also tend to be increased in the subjects with higher values of mRSS. Main text Figure 1c plots association with mRSS on log base 2 scale per one unit of mRSS versus log base 2 SSc/Cont ratio, labels indicate serum proteins with BH-FDR<0.05 in both comparisons.

Statistical significance of this correlation was evaluated with permutation by repeatedly ($N = 9999$ permutations) fitting corresponding linear models for the dataset with randomly reassigned sample annotations and molecular profiles, and calculating rank correlations between resulting model parameter estimates.

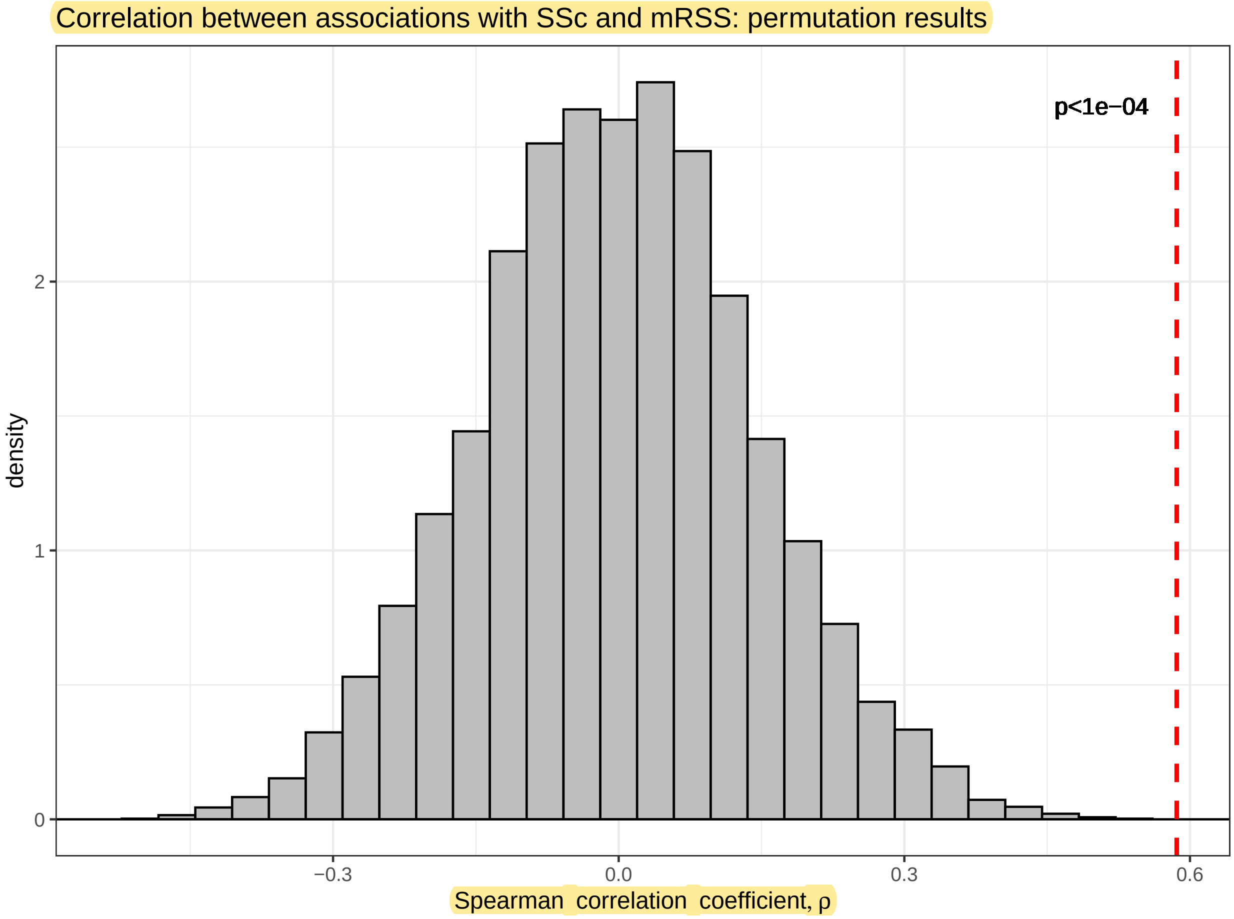

Figure S3: Permutation significance of the correlation between SSc-Cont differences in serum proteins and their associations with mRSS. Vertical red dashes and histogram represent the Spearman correlation coefficient observed for the original sample annotation and corresponding null distribution of their values for randomly permuted sample annotation, respectively.

Comparison of the correlation observed for the original sample annotation (vertical red dashes in Supplementary Figure S3) to the null distribution obtained from repeating this analysis with randomly permuted sample annotation (shown as histogram in Supplementary Figure S3) reveals that the observed correlation coefficient has not been observed across the permutation study (p<1e-04).

5 Comparison of SSc-Cont differential expression in the serum, PBC and skin

The differences between SSc and Cont groups observed for serum proteins were correlated with those observed for corresponding transcripts in skin and PBC datasets. The entire collection of serum proteins characterized by Olink and available for differential expression analysis were mapped to PBC (for serum-PBC comparison, 314 matching analytes) or to skin (for serum-skin comparison, 448 matching analytes) gene expression data

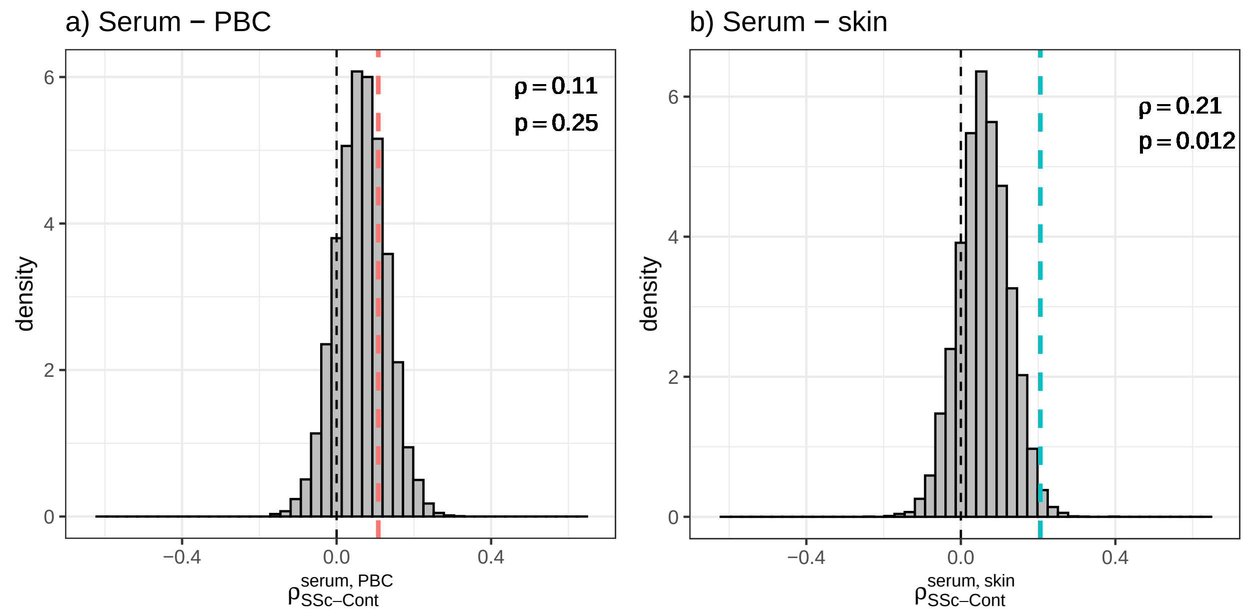

Figure S4: Correlations between SSc-Cont differences for serum proteins and corresponding transcripts in PBC (a) and skin (b). Histograms represent null distributions obtained for permuted sample annotations, vertical dashes – those observed for original SSc-Cont status of the samples.

by their Entrez Gene identifiers. SSc-Cont differences for serum proteins, PBC and skin transcripts were calculated adjusting for covariates as described above. The rank (Spearman) correlation between SSc-Cont differences for skin transcripts and corresponding serum proteins was 0.21 and 0.11 for PBC transcripts and serum proteins. Statistical significance of these correlations was estimated by permutations ($N = 9999$), repeating these calculations for sample annotations with randomized SSc/Cont status of study subjects. As illustrated by Supplementary Figure S4, the frequencies of rank correlations observed in permutation controls equal to or exceeding (by absolute magnitude for two-sided p-value) those obtained for the original sample annotation yielded $p = 0.012$ for correlation of SSc-Cont differences between serum proteins and skin transcripts and $p = 0.25$ for correlation between serum proteins and PBC transcripts.

6 Pathway connectedness between differentially expressed transcripts and proteins

The recently published well-associated protein (WAP) methodology (7) was employed to obtain rankings of genes by their WAP scores for the differential expression in SSc and Cont groups for PBC and skin gene expression datasets. This produced two orderings (for PBC and for skin data) of all genes in the pathway network by their connectedness to the genes that are more dysregulated at the transcriptional level between SSc and Cont. Then, given the ordering of serum proteins by their significance in SSc-Cont comparison, average ranks of the corresponding pathway nodes by their WAP scores with PBC and skin data were calculated and compared for the top 1, 2, . . . , etc. up to all serum proteins measured by Olink that are present in pathway network. The lower ranks of WAP scores represent greater significance of their connectedness on pathway network to more dysregulated transcripts in SSc-Cont comparison, and lower average ranks of WAP scores indicate more significant connectedness of serum proteins to corresponding transcripts on pathway network. Statistical significance of the resulting difference can be evaluated by comparing observed differences to their null distribution obtained from repeating this analysis for the transcriptional and proteomic data with randomly permuted sample annotation data. The network of functional relationships between proteins

(genes) utilized for this analysis was derived from STRING v.10 (8) by restricting interactions to those with their confidence score of at least 0.7 (9). Detailed description of WAP methodology and analysis of its performance in the context of the analysis of gene expression data from human translational studies can be found in (7).

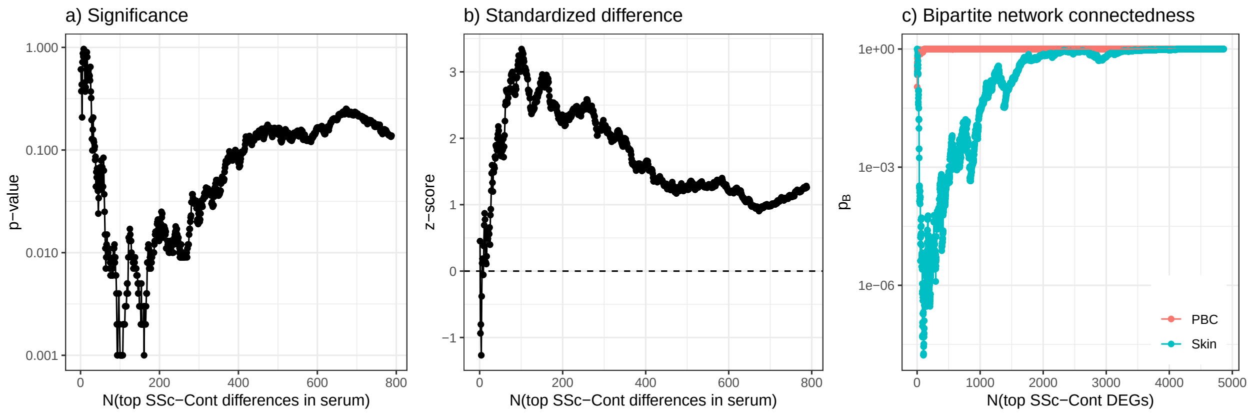

Figure S5: The serum proteins that are more significantly different between SSc and Cont groups are also more significantly connected on pathway network to the genes more significantly different in SSc vs. Cont comparison in skin as compared to those differences in PBC.

Main text Figure 3, panel b) and Supplementary Figure S6 provide detailed representation of this analysis for the serum proteins that are differentially expressed (BH-FDR<0.05) between SSc and Cont groups. Supplementary Figure S5 (panels a-b) displays results of this analysis for the entire set of serum proteins characterized in this study. Panel a) represents permutation estimates of the significance of the difference between average ranks of WAP scores of SSc-Cont comparison in PBC vs. corresponding ranks in skin for the top 1, 2, . . . , N serum proteins in the order of their decreasing statistical significance in SSc-Cont comparison. The highest significance (p<0.001) of such difference between mean WAP score ranks in PBC and skin is observed for about 100 serum proteins most significantly different between SSc and Cont. Panel b) represents the difference between mean ranks of WAP scores in PBC and skin for corresponding sets of serum proteins in the order of their decreasing significance of SSc-Cont differences normalized to their mean and standard deviation in permutation controls. Positive values represent higher, less significant ranks of WAP scores in the SSc-Cont differential gene expression analysis in PBC than in skin. Main text Figure 2, panel c) depicts edge-count significance of the network connections between serum proteins differentially expressed between SSc and Cont and several numbers (top 50, 100 and 200) of the most significantly differentially expressed transcripts in PBC and skin. Panel c) in Supplementary Figure S5 displays values of edge-count significance of connections between differentially expressed serum proteins and broader range of DEGs in PBC and skin. Across a large range of SSc-Cont differences at the transcript level, serum proteins differentially expressed between SSc and Cont are several orders of magnitude more significantly connected to differentially expressed transcripts in skin as compared to PBC.

7 Comparison of similarities among molecular profiles of skin, PBC and serum

7.1 Correlations between transcripts in PBC and skin and serum proteins

Transcriptional profiles of PBC samples and skin biopsies obtained concomitantly with serum proteomic data for the same patients with systemic sclerosis and healthy controls allow evaluation of correlation between these molecular measurements at the level of individual genes/proteins as well as assessment of the concordance of

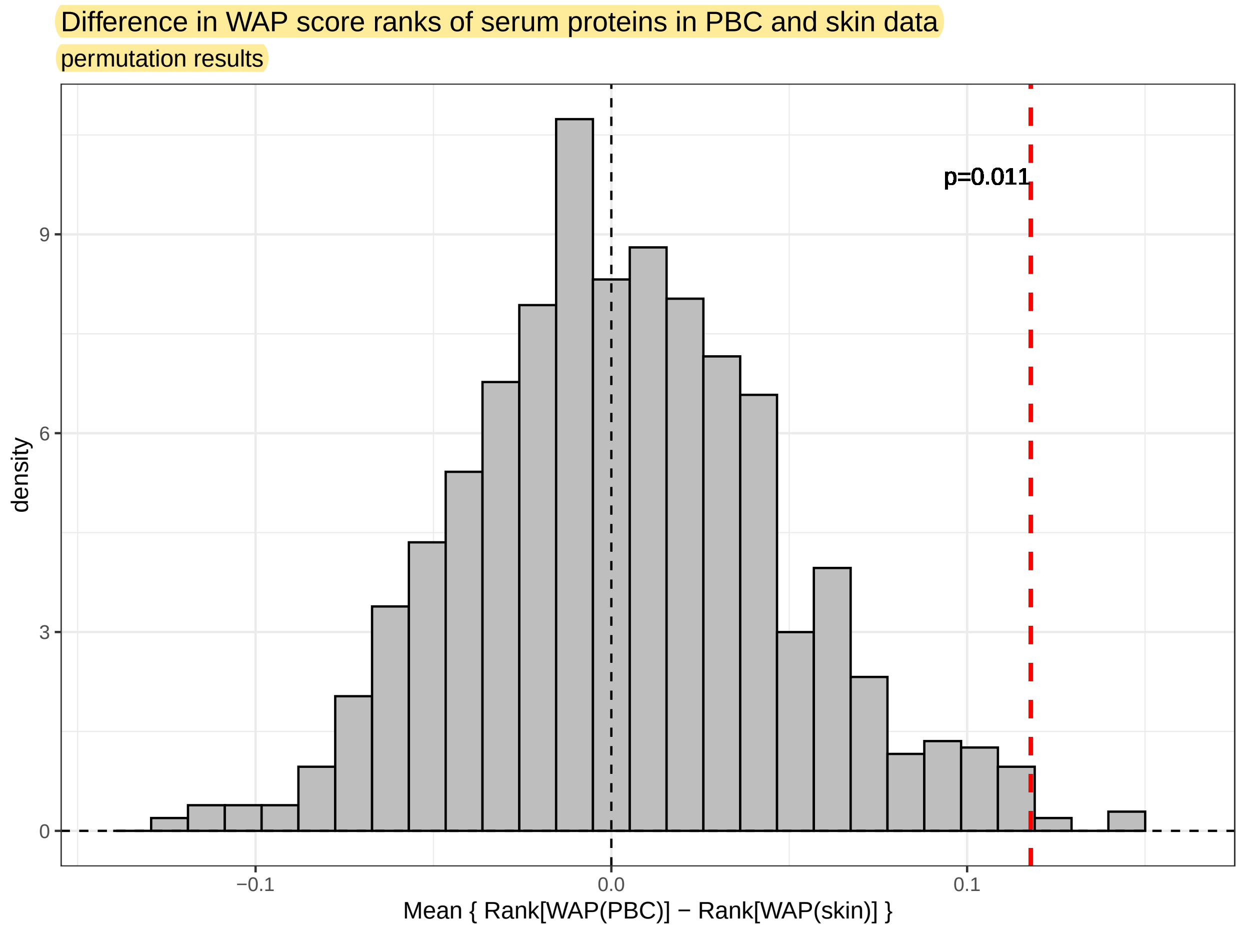

Figure S6: Permutation significance of the difference between average ranks of serum proteins differentially expressed in SSc in WAP analysis of SSc-Cont differences in PBC and skin data. Vertical red dashes indicate observed difference between average ranks of serum proteins WAP scores in PBC and skin. Histogram represents corresponding null distribution of the differences obtained from the analysis of the data conducted with randomly permuted sample annotation. Positive values represent greater significance of serum proteins as WAPs in skin than in PBC.

between-sample similarities when calculated for these entire datasets. For the pairwise comparison of Illumina probes expression levels in PBC and skin transcriptional data they were limited to 7206 of those with average detection p-value below 0.01 both in PBC and skin gene expression datasets. For the comparisons of PBC and skin transcriptional profiles to serum proteomic data gene-level average expression profiles were obtained for Illumina probes with average detection p-value below 0.01 in PBC and skin respectively.

A subset of Illumina probes displays correlation between their expression levels (adjusted for the effects of sample collection date and Illumina chip identity) in PBC and skin samples both in patients with systemic sclerosis as well as in healthy controls in excess of what would be expected for randomly matched skin and PBC samples at FDR<5% as estimated by permutation. Supplementary Figures S7 through S11 display XY-scatterplots of these probes' intensities in PBC (horizontal axis) and skin (vertical axis) gene expression data with cue shape and color representing subject gender (male or female) and study group membership (patients with SSc or healthy controls) respectively. Numerical values of the corresponding correlation coefficients are shown in Supplementary Table S8 in the supplementary data file `Additional_File_2.xlsx`. Several of these probes are mapped to the same ribosomal protein S26 (RPS26) and display comparable amount of positive correlation between their levels in PBC and skin data both in patients with systemic sclerosis and healthy controls with a subset of subjects measuring substantially (one or two units on log base 2 scale) lower than the rest of them. Similarly, comparable levels of positive correlation between skin and PBC expression levels in subjects with systemic sclerosis and healthy controls are observed for the majority of the probes (genes) displayed in these plots, with a few exceptions (e.g. probes mapped to genes TCHP, TRPT1, NFKBIA and MT1X) where the correlation between skin and PBC appears to differ between SSc and Cont groups. Additionally, the correlations between the following serum proteins and corresponding PBC transcripts are positive both in SSc and Cont groups and pass 5% threshold on permutation-estimated FDR: FCRL3, FOLR3, GZMH, LILRA5, TREM1.

7.2 Mantel test-based evaluation of the concordances of between-sample similarities

Consider n study subjects characterized by gene expression measurements on their PBC and skin samples b_{ij} and s_{ik}, $1 \leq i \leq n$, $1 \leq j \leq p_b$, $1 \leq k \leq p_s$ for p_b and p_s genes measured in PBC and skin respectively with corresponding vectors of PBC $\mathbf{b}_i \in \mathbb{R}^{p_b}$ and skin $\mathbf{s}_i \in \mathbb{R}^{p_s}$ gene expression measurements for the same study subject i appearing with the same index i both in PBC and skin datasets. Dissimilarity of PBC gene expression data for two study subjects l and m can be quantified as one-complement of Pearson correlation coefficient $d^{(b)}_{lm} = 1 - \mathrm{cor}(\mathbf{b}_l, \mathbf{b}_m)$ between their respective vectors $\mathbf{b}_l$ and $\mathbf{b}_m$ of gene expression measurements in PBC (and, similarly, $d^{(s)}_{lm} = 1 - \mathrm{cor}(\mathbf{s}_l, \mathbf{s}_m)$ for skin), where $\mathrm{cor}(\mathbf{x}, \mathbf{y}) = \frac{\sum(x_i - \bar{x})(y_i - \bar{y})}{\sqrt{\sum(x_i - \bar{x})^2}\sqrt{\sum(y_i - \bar{y})^2}}$, $\bar{a} = \sum_{i=1}^{q} a_i/q, \mathbf{a} \in \mathbb{R}^q$. Resulting dissimilarities d_{lm} equal to zero when the measurements for the two samples are perfectly correlated, so that $\mathrm{cor}(\mathbf{x}_l, \mathbf{x}_m) = 1$, are closely related to Euclidean distance: $1 - \mathrm{cor}(\mathbf{x}^*, \mathbf{y}^*) = \frac{\|\mathbf{x}^* - \mathbf{y}^*\|^2}{2p}$, $\|\mathbf{a}\| = \sqrt{\sum_{i=1}^{q} a_i^2}, \mathbf{a} \in \mathbb{R}^q$ when $\mathbf{x}$ and $\mathbf{y}$ in $\mathbb{R}^p$ have been standardized, so that $\bar{x^*} = \bar{y^*} = 0$, $\|x^*\| = \|y^*\| = \sqrt{p}$, and can be calculated on the ranks of the gene expression measurements, thereby switching to Spearman correlation coefficient, to decrease the impact of the more variable observations.

Between-sample dissimilarities in PBC $\mathbf{b}_i$ and skin $\mathbf{s}_i$ gene expression measurements for all $n(n-1)/2$ pairwise combinations of n samples can be represented by two $n \times n$ matrices $\mathbf{D}^{(b)}$ and $\mathbf{D}^{(s)}$ respectively. This representation enables further assessment of the concordance among between-sample dissimilarities in PBC and skin data by employing permutation controls as outlined in (10) to statistically access whether samples that are more similar when comparing their gene expression measurements in PBC also tend to be more similar comparing their gene expression profiles in skin.

Briefly, the test statistic quantifying such concordance of between-sample dissimilarities in PBC and skin data now can be defined as the correlation between non-diagonal elements in dissimilarity matrices $\mathbf{D}^{(b)}$ and $\mathbf{D}^{(s)}$:

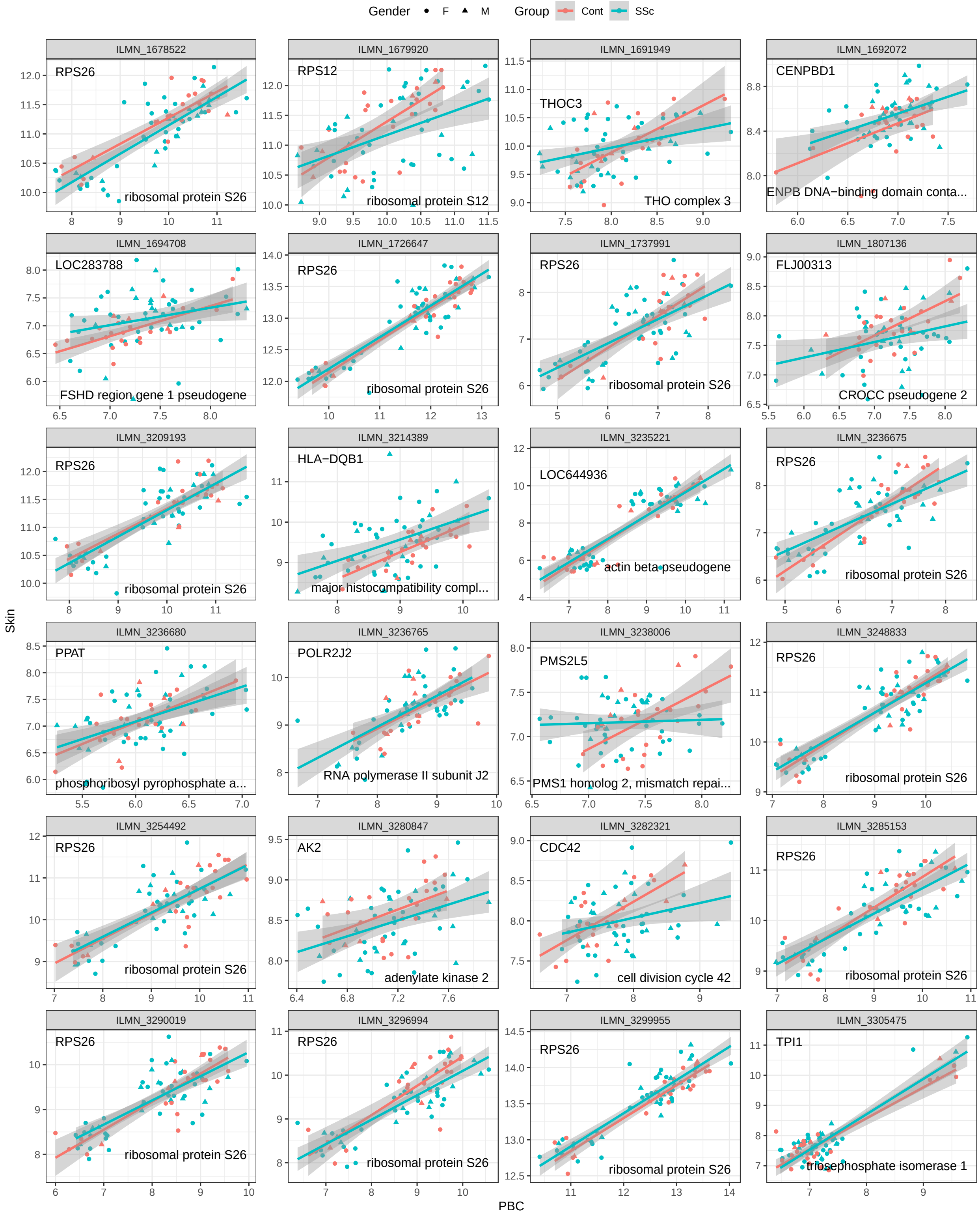

Figure S7: Top 1-24 most correlated probes between skin and PBC in subjects with systemic sclerosis and healthy controls.

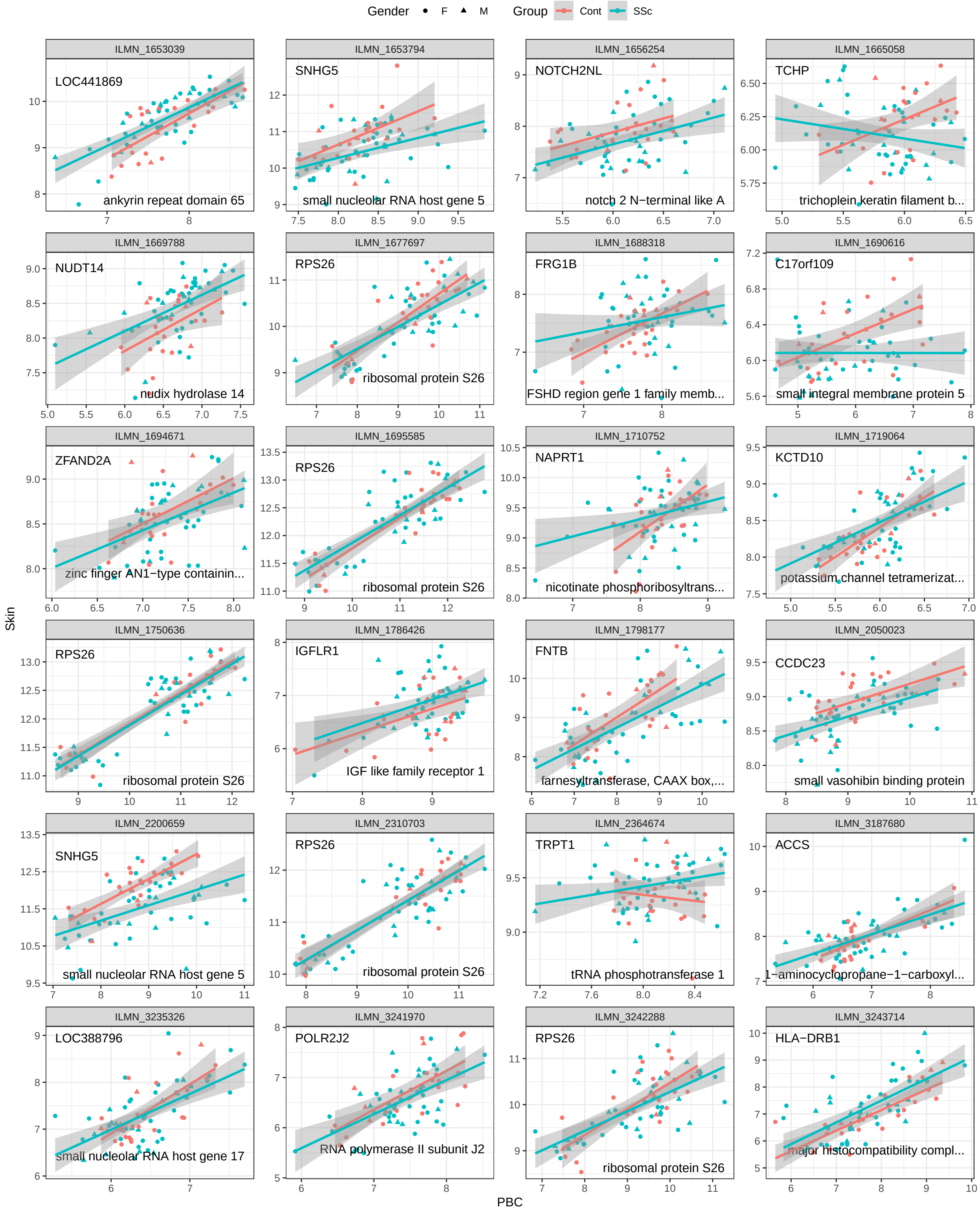

Figure S8: Top 25-48 probes most correlated between skin and PBC in subjects with systemic sclerosis and healthy controls.

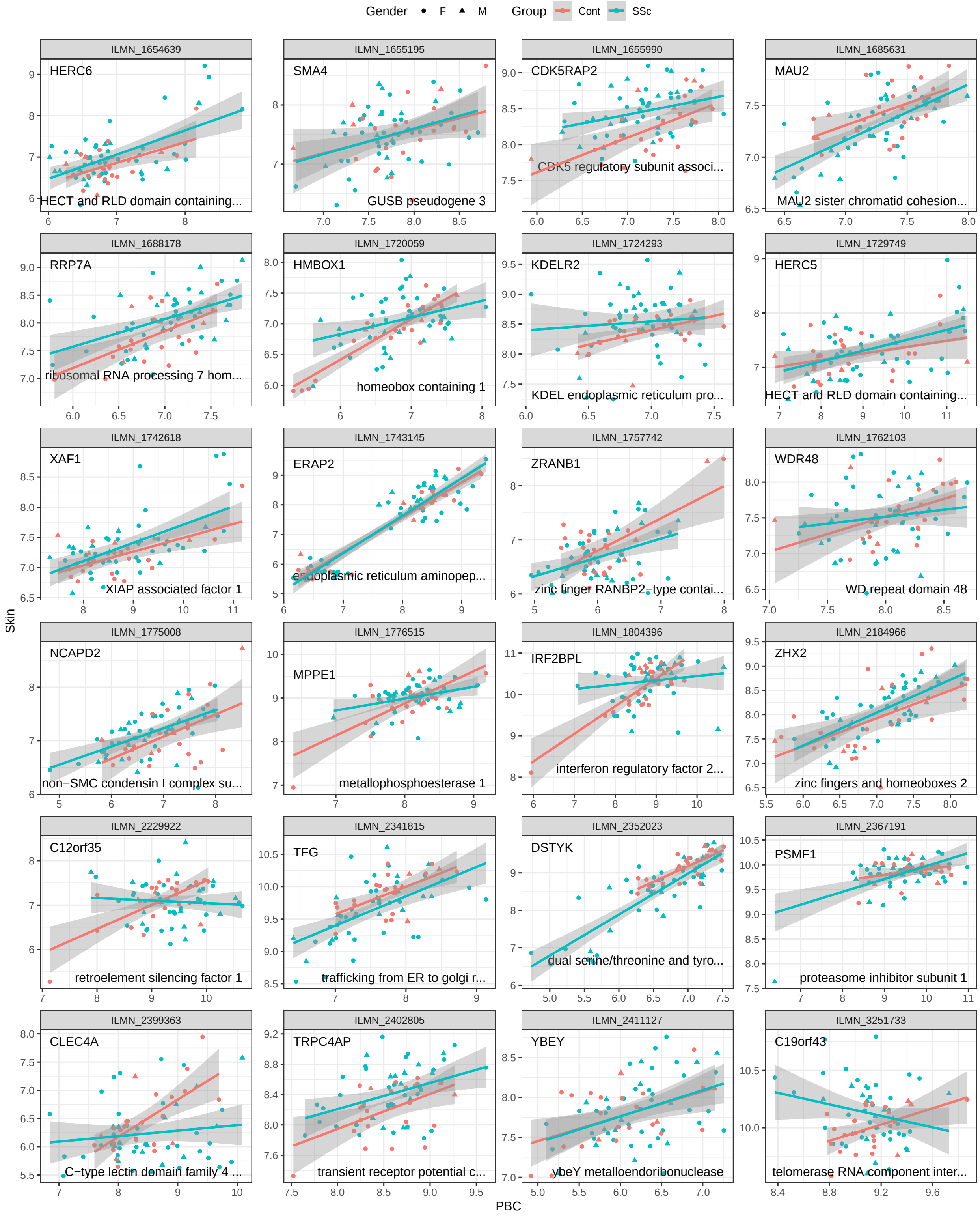

Figure S9: Top 49-72 probes most correlated between skin and PBC in subjects with systemic sclerosis and healthy controls.

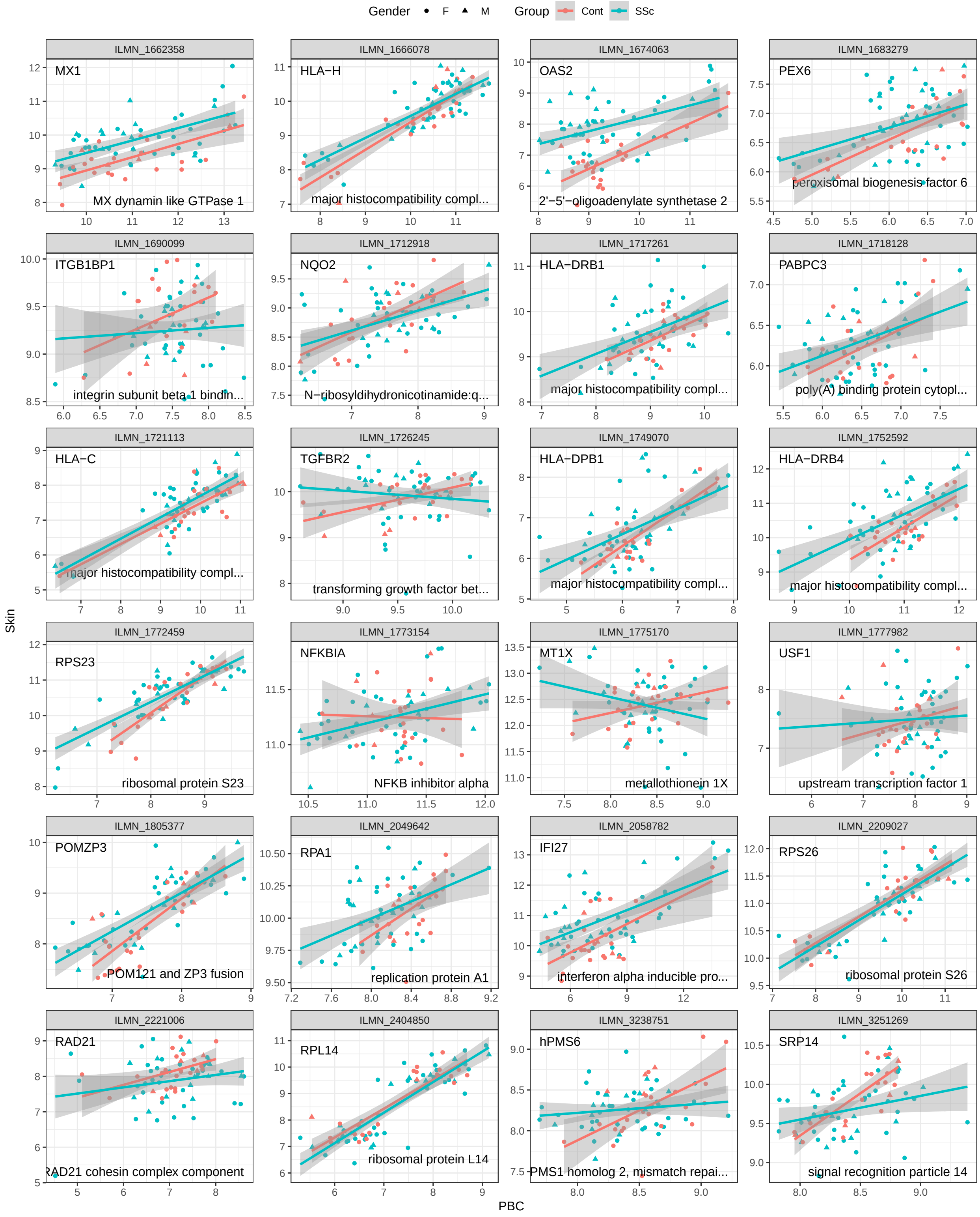

Figure S10: Top 73-96 probes most correlated between skin and PBC in subjects with systemic sclerosis and healthy controls.

$$r_{bs}^{\mathbf{D}} = \mathrm{cor}\left(\mathbf{D}^{(b)}, \mathbf{D}^{(s)}\right) = \frac{\sum_{l<m}^{n}\left(d_{lm}^{(b)} - \bar{d}^{(b)}\right)\left(d_{lm}^{(s)} - \bar{d}^{(s)}\right)}{\sqrt{\sum_{l<m}^{n}\left(d_{lm}^{(b)} - \bar{d}^{(b)}\right)^2}\sqrt{\sum_{l<m}^{n}\left(d_{lm}^{(s)} - \bar{d}^{(s)}\right)^2}}$$

where $\bar{d}^{(b)} = 2\sum_{l<m}^{n} d_{lm}^{(b)} / \left(n^2 - n\right)$ and $\bar{d}^{(s)} = 2\sum_{l<m}^{n} d_{lm}^{(s)} / \left(n^2 - n\right)$ are average between-sample dissimilarities in PBC and skin data respectively. Simple extension of this procedure outlined in (10) is to replace $d_{lm}^{(b)}$ and $d_{lm}^{(s)}$ in the above equation with their respective ranks, effectively calculating Spearman instead of Pearson correlation above.

Statistical significance of the observed PBC-skin concordance of between-samples dissimilarities, $r_{bs}^{\mathbf{D}}$, can be evaluated by comparing it to its null distribution that can be obtained by randomly permuting matching sample assignment in PBC and/or skin data that can accomplished by randomly reordering *rows and corresponding columns*, to account for the dependencies among all dissimilarities involving the same samples as emphasized in (10), in dissimilarity matrices $\mathbf{D}^{(b)}$ and $\mathbf{D}^{(s)}$ and using resulting permuted matrices $\tilde{\mathbf{D}}^{(b)}$ and $\tilde{\mathbf{D}}^{(s)}$ to calculate $R_{bs}^{\mathbf{D}} = \mathrm{cor}\left(\tilde{\mathbf{D}}^{(b)}, \tilde{\mathbf{D}}^{(s)}\right)$ over a sufficiently large number of permutations. Low values of the resulting $\Pr\left[R_{bs}^{\mathbf{D}} \geq r_{bs}^{\mathbf{D}}\right]$ indicate that for the samples that are more similar to each other in PBC gene expression data their corresponding transcriptional profiles in skin also tend to be more similar as compared to randomly paired gene expression profiles in PBC and skin datasets. Trivial change in notation adapts this example to the comparisons of between-subject dissimilarities in PBC or skin transcriptional profiling datasets to those in serum proteomic data.

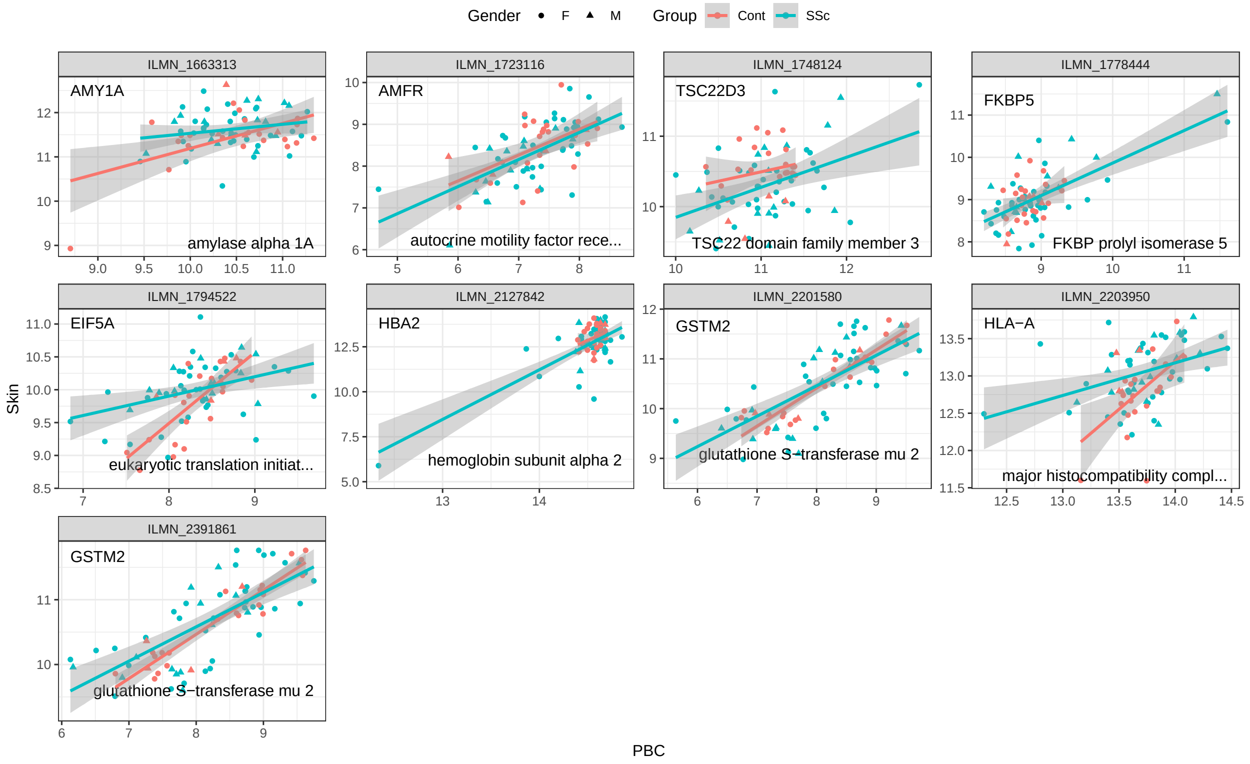

Figure S11: Top 97-105 probes most correlated between skin and PBC in subjects with systemic sclerosis and healthy controls.

Application of Mantel test (10) as implemented by `ade4::mantel.randtest` in R (11) for assessing concordance of similarities between matching observations as calculated by two different metrics (e.g. similarities

in PBC and in skin gene expression data) with respect to permutation control enables estimation of the significance the similarities between samples for the entire sets of gene expression measurements in skin and PBC as well as protein levels in serum. Figure 4 in the main text summarizes results of this evaluation in the form of histograms of similarities observed in permutations and those observed for original matching of molecular profile for SSc and Cont subjects for each pairwise combination of PBC transcriptomic, skin transcriptomic and serum proteomic profiles.

Session Info

The information below represents versions of R / Bioconductor statistical software used to generate computational results presented above.

- R version 4.0.2 (2020-06-22), `x86_64-w64-mingw32`
- Locale: `LC_COLLATE=English_United States.1252`, `LC_CTYPE=English_United States.1252`, `LC_MONETARY=English_United States.1252`, `LC_NUMERIC=C`, `LC_TIME=English_United States.1252`
- Running under: `Windows 10 x64 (build 18363)`
- Matrix products: default
- Base packages: base, datasets, graphics, grDevices, methods, parallel, stats, stats4, utils
- Other packages: ade4 1.7-17, AnnotationDbi 1.52.0, Biobase 2.50.0, BiocGenerics 0.36.1, doParallel 1.0.16, doRNG 1.8.2, foreach 1.5.1, ggplot2 3.3.4, ggrepel 0.9.1, graph 1.68.0, gridExtra 2.3, illuminaHumanv4.db 1.26.0, IRanges 2.24.1, iterators 1.0.13, kableExtra 1.3.4, limma 3.46.0, MASS 7.3-51.6, Matrix 1.2-18, msigdbr 7.4.1, org.Hs.eg.db 3.12.0, plyr 1.8.6, ppcor 1.1, reshape2 1.4.4, rngtools 1.5, S4Vectors 0.28.1, SparseM 1.81, xlsx 0.6.5
- Loaded via a namespace (and not attached): babelgene 21.4, bit 4.0.4, bit64 4.0.5, bitops 1.0-7, blob 1.2.1, bookdown 0.22, cachem 1.0.5, caTools 1.18.2, codetools 0.2-16, colorspace 2.0-1, compiler 4.0.2, crayon 1.4.1, DBI 1.1.1, digest 0.6.27, dplyr 1.0.6, ellipsis 0.3.2, evaluate 0.14, fansi 0.5.0, farver 2.1.0, fastmap 1.1.0, generics 0.1.0, glue 1.4.2, gplots 3.1.1, grid 4.0.2, gtable 0.3.0, gtools 3.9.2, highr 0.9, htmltools 0.5.1.1, httr 1.4.2, KernSmooth 2.23-17, knitr 1.33, labeling 0.4.2, lattice 0.20-41, lifecycle 1.0.0, magrittr 2.0.1, memoise 2.0.0, mgcv 1.8-31, munsell 0.5.0, nlme 3.1-148, pillar 1.6.1, pkgconfig 2.0.3, purrr 0.3.4, R6 2.5.0, Rcpp 1.0.6, rJava 1.0-4, rlang 0.4.11, rmarkdown 2.9, RSQLite 2.2.7, rstudioapi 0.13, rvest 1.0.0, scales 1.1.1, sfsmisc 1.1-11, splines 4.0.2, stringi 1.5.3, stringr 1.4.0, svglite 2.0.0, systemfonts 1.0.2, tibble 3.1.2, tidyselect 1.1.1, tools 4.0.2, utf8 1.2.1, vctrs 0.3.8, viridisLite 0.4.0, webshot 0.5.2, withr 2.4.2, xfun 0.24, xlsxjars 0.6.1, xml2 1.3.2, yaml 2.2.1

Elapsed compilation time: 3417.51 sec.

Supplementary References

1. D'Alessandro JS, Duffner J, Pradines J, Capila I, Garofalo K, Kaundinya G, et al. Equivalent gene expression profiles between glatopa and copaxone. *PloS one* 2015;10:e0140299.

2. Sobolev O, Binda E, O'farrell S, Lorenc A, Pradines J, Huang Y, et al. Adjuvanted influenza-h1n1 vaccination reveals lymphoid signatures of age-dependent early responses and of clinical adverse events. *Nature immunology* 2016;17:204.

3. Wu D, Smyth GK. Camera: A competitive gene set test accounting for inter-gene correlation. *Nucleic acids research* 2012;40:e133–e133.

4. Uhlen M, Karlsson MJ, Zhong W, Tebani A, Pou C, Mikes J, et al. A genome-wide transcriptomic analysis of protein-coding genes in human blood cells. *Science* 2019;366.

5. Swindell WR, Johnston A, Voorhees JJ, Elder JT, Gudjonsson JE. Dissecting the psoriasis transcriptome: Inflammatory-and cytokine-driven gene expression in lesions from 163 patients. *BMC genomics* 2013;14:527.

6. Assassi S, Swindell WR, Wu M, Tan FD, Khanna D, Furst DE, et al. Dissecting the heterogeneity of skin gene expression patterns in systemic sclerosis. *Arthritis & Rheumatology* 2015;67:3016–3026. Available at: https://onlinelibrary.wiley.com/doi/abs/10.1002/art.39289.

7. Pradines JR, Farutin V, Cilfone NA, Ghavami A, Kurtagic E, Guess J, et al. Enhancing reproducibility of gene expression analysis with known protein functional relationships: The concept of well-associated protein. *PLoS computational biology* 2020;16:e1007684.

8. Szklarczyk D, Franceschini A, Wyder S, Forslund K, Heller D, Huerta-Cepas J, et al. STRING v10: protein-protein interaction networks, integrated over the tree of life. *Nucleic Acids Res* 2015;43:D447–52.

9. Mering C von, Jensen L, Snel B, Hooper S, Krupp M, Foglierini M, et al. STRING: known and predicted protein-protein associations, integrated and transferred across organisms. *Nucleic Acids Res* 2005;33:D433–7.

10. Mantel N. The detection of disease clustering and a generalized regression approach. *Cancer research* 1967;27:209–220.

11. Chessel D, Dufour AB, Thioulouse J, others. The ade4 package-i-one-table methods. *R news* 2004;4:5–10.
